# SPITFIR(e): a supermaneuverable algorithm for fast denoising and deconvolution of 3D fluorescence microscopy images and videos

**DOI:** 10.1038/s41598-022-26178-y

**Published:** 2023-01-27

**Authors:** Sylvain Prigent, Hoai-Nam Nguyen, Ludovic Leconte, Cesar Augusto Valades-Cruz, Bassam Hajj, Jean Salamero, Charles Kervrann

**Affiliations:** 1 SERPICO Project-Team, Inria Centre Rennes-Bretagne Atlantique, 35042 Rennes Cedex, France; 2 SERPICO/STED Team, UMR144 CNRS Institut Curie, PSL Research University, Sorbonne Universités, 75005 Paris, France; 3 Laboratoire Physico-Chimie, Institut Curie, PSL Research University, Sorbonne Universités, CNRS UMR168, 75005 Paris, France

**Keywords:** Image processing, Imaging, Microscopy, Software, Cellular imaging

## Abstract

Modern fluorescent microscopy imaging is still limited by the optical aberrations and the photon budget available in the specimen. A direct consequence is the necessity to develop flexible and “off-road” algorithms in order to recover structural details and improve spatial resolution, which is critical when restraining the illumination to low levels in order to limit photo-damages. Here, we report SPITFIR(e) a flexible method designed to accurately and quickly restore 2D–3D fluorescence microscopy images and videos (4D images). We designed a generic sparse-promoting regularizer to subtract undesirable out-of-focus background and we developed a primal-dual algorithm for fast optimization. SPITFIR(e) is a ”swiss-knife” method for practitioners as it adapts to any microscopy techniques, to various sources of signal degradation (noise, blur), to variable image contents, as well as to low signal-to-noise ratios. Our method outperforms existing state-of-the-art algorithms, and is more flexible than supervised deep-learning methods requiring ground truth datasets. The performance, the flexibility, and the ability to push the spatiotemporal resolution limit of sub-diffracted fluorescence microscopy techniques are demonstrated on experimental datasets acquired with various microscopy techniques from 3D spinning-disk confocal up to lattice light sheet microscopy.

## Introduction

Fluorescence microscopy provides a very powerful framework to biologists for observing, analyzing, and studying specific fluorescently-tagged structures and biological processes at very high spatial and temporal resolutions. Despite number of advantages, there are two major limitations of fluorescence microscopy. The first limitation is the presence of photon (shot) noise in acquired images. Shot noise is mainly due to the quantum nature of light, implying that the arrival of a photon on a sensor is a random event and thus the number of incident photons over a period of time is a random variable depending on the brightness of the light source. Moreover, in biological samples the signal-to-noise-ratio (SNR) is usually very low because low dose of illumination light is required to avoid photo-bleaching (i.e., progressive fading of the emission intensity) of fluorescent molecules and to preserve specimen integrity (photo-toxicity)^1,2^. Additionally, the quality of acquired fluorescence images is worsened by the blurring effects induced by several factors such as excitation wavelength, immersion medium refraction, specimen thickness, and the limited aperture of the objective which results in light diffraction through the optical system. The diffraction phenomenon implies that light emitted by an infinitely small point source appears wider at the focal plan and spreads into a specific pattern called “point spread function” (PSF).

Noise and blur not only degrade the image quality in terms of overall visualization but also have a negative influence on specimen analysis, including detection and segmentation. To improve the quality of images acquired by fluorescence microscopes, restoration (deconvolution and/or denoising) is then frequently applied as pre-processing step before quantitative analysis, and amounts to recovering the original unknown image *u* from the observed noisy and blurred image *f* represented as follows: $f = {{\mathcal {T}}}(h *u)$, where *h* denotes the 2D or 3D spatial response of the device representing the blur related to the optical system (e.g., PSF) assumed to be linear shift-invariant, $*$ denotes the convolution operator, and ${{\mathcal {T}}}$ is a degradation operator modeling the measurement noise. If the image is not blurred but mainly corrupted by noise, *h* is represented by the Dirac (or impulse) function and the restoration problem translates into a denoising problem. Finally, the degradation operator (shot noise and readout noise) is usually modeled as a mixed Poisson-Gaussian distribution which is non-stationary and signal-dependent.

In the last twenty years, many restoration methods have been investigated in order to “inverse” the model in 2D-3D fluorescence microscopy. The most popular restoration approaches are linear methods (e.g., Wiener filtering). Despite the simplicity and low-computation-requirement, they usually do not restore fine image details at frequency components that are beyond the bandwidth of the PSF (i.e., the support of its Fourier transform). Another popular technique is the Richardson-Lucy (RL) algorithm^3–5^. While well-dedicated to Poisson noise removal, RL deconvolution amplifies and creates structural noise and artifacts after a small number of iterations, which constitutes a major problem in fluorescence imaging^6^. To reduce these drawbacks and iteration number, several modern and fast RL implementations has been recently proposed^7,8^. Actually, the most flexible and robust deconvolution methods consist in minimizing an energy functional composed of a data fidelity term and a regularization term that encompasses prior knowledge (positivity, smoothness, sparsity,...) about the solution. The seminal Tikhonov-Miller (TM) approach^9–11^ may be considered as the starting point to the development of regularization methods in fluorescence microscopy. While it was successful in many applications, the TM regularizer tends to produce blurred images since low gradient magnitudes are encouraged in the entire image, including at image discontinuities. To avoid over-smoothing caused by quadratic functionals, non-quadratic regularizers have been first studied, especially the Total Variation (TV) regularizer that penalizes the $L_1$ norm of the gradient. Nevertheless, TV creates “stair-casing” effects, which are particularly undesirable in fluorescence imaging^12,13^. To overcome this disadvantage, Lefkimmiatis *et al.*^14^ recently proposed a family of convex regularizers built upon the matrix norm of the Hessian (Schatten norm), computed at each point of the image. This piece-wise smoothness promoting regularizer combined with a non-quadratic data term, was recently evaluated in fluorescence microscopy^15^. Nevertheless, this regularizer does not impose sparsity prior on the fluorescent signals which is also an important feature in fluorescence imaging. This idea was investigated by Arigovindan *et al. *^16^ who reported good results 3D wide-field fluorescence images by using a non-convex sparsity-promoting regularizer; this regularizer (named here Log Sparse Hessian Variation (LHSV)) has been implemented into the entropy-regularized deconvolution algorithm (ER-Decon). Concurrently, other sparsity-promoting methods, focused on first-order differentiation-based regularizers have been investigated in medical imaging such as GraphNet (GN)^17–19^ and Sparse Variation (SV)^20^, and in image processing (e.g., TV-${L_1}$^21,22^). Nevertheless, the sparsity-promoting regularizers reported in Table 2 have limitations. They were designed for 2D medical imaging^17–20^ or 3D microscopy^16,23^ but never for both. Moreover, they are based on the first-order^17–20^ or second derivatives^16,23^, and are formulated as non-convex^16^ or convex^23^ optimization problems. While more tricky, non-convex energies are now efficiently minimized by performant algorithms such as ADMM^24,25^ and FISTA^26^.

To overcome the main limitations of previous methods, we introduce a fast, supermaneuverable method (SPITFIR(e)) to deconvolve and/or denoise 2D or 3D multispectral fluorescence microscopy images and videos. Our method outperforms existing algorithms in three ways. (1) SPITFIR(e) includes a generic sparsity promoting regularizer to discard out-of-focus background, and adapts to most if not all fluorescence microscopy techniques even if the 3D PSF is not well known. (2) It reaches high restoration performance for variable levels and sources of noise and blur. It outperforms state-of-the-art deconvolution methods and is competitive when compared to supervised deep-learning methods. (3) The SPITFIR(e) parameters are automatically calibrated from the image contents and the practitioner needs only to specify if the desired image is “highly”, “moderately” or “weakly” sparse. A “weakly” sparse image contains clutter and complex contents. We validated the overall performance of our approach on several challenging images acquired with lattice light sheet (LLSM), stimulated emission depletion (STED), multifocus microscopy (MFM), and spinning-disk confocal microscopy (CM). For the evaluation on different imaging techniques, we have chosen to apply SPITFIR(e) on a limited number of structures relatively difficult to optically resolve or for which the photon budget is critical, i.e., mitochondria, cytoskeleton elements and biomolecules associated to moving vesicles in living cells. We also developed CPU and GPU implementations which can be downloaded as open-source from the GitHub website (https://github.com/sylvainprigent/spitfire) under a AGPL-3.0 license. Finally SPITFIR(e) is interfaced with napari for ease of use (https://www.napari-hub.org/plugins/napari-sdeconv).

**Figure 1:**
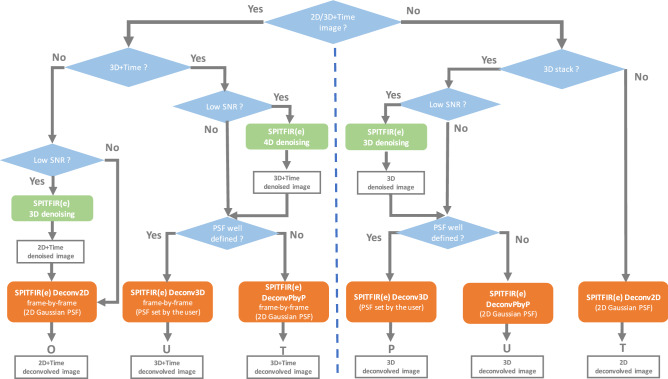
SPITFIR(e) overview. The flow chart describes the sequence of restoration steps applied to 3D, 2D+Time, or 3D+Time images based on responses of end-users.

## Results

### Overview of SPITFIR(e)

SPITFIR(e) is an algorithm well-grounded in the regularization theory for inverse problems, that can robustly recover information from general multidimensional noisy and blurry fluorescence images. Unlike the traditional methods requiring the tedious manual adjustment of the parameter that balances the data fidelity term and the prior term, SPITFIR(e) includes a self-tuning and scale-invariant technique for automatic adaptation ("Methods"). To our knowledge, there are few methods able to select such parameters in the literature to process complex 3D microcopy images. Here, we mention an alternative approach^27^ that exploits a single image and subsampling operations to compute Fourier Ring Correlation and further to determine restoration algorithm parameters and assess the most appropriate spatial resolution. In our approach, the practitioner can modify the default parameter setting and encourage more sparsity and/or smoothness from a preview of a $3 \times 3$ matrix depicting the restoration results on a 2D ROI with three levels – “weak”, “moderate”, “high” – of sparsity and smoothness. The minimization of the sum of the two convex terms is originally implemented in a computationally efficient way with a recent version of proximal-splitting optimization algorithm^28,29^ (see Methods and Appendix). In what follows, we show that SPITFIR(e) can restore 3D fluorescence images under low excitation light excitation conditions by chaining denoising and deconvolution with the same algorithm with very small computing times compared to other competitive methods^15,23^.

### SPITFIR(e) is supermaneuverable and adapts to every fluorescence microscopy modality

SPITFIR(e) is said ”supermaneuverable” as it includes several strategies to adapt to microscopy specificities, and to particular spatial and temporal acquisition conditions (see flowchart in Fig. 1). For instance, the practitioner can apply the conventional 3D deconvolution strategy if the PSF is perfectly known. If this is not the case, a “plane by plane” (PbyP) strategy which consists in separately deconvolving each XY section of the 3D stack given a 2D Gaussian PSF model, can be applied. Surprisingly, the PbyP strategy could produce better visual results and is faster than direct 3D deconvolution with an imperfect or approximately measured 3D PSF. Furthermore, in the case of low-photon regimes or low-exposure times, the results may be significantly improved by applying the “denoise-before-deconvolve” approach. For instance, a 3D+Time (4D) volume can be denoised as a whole, while the planes of each 3D stack are independently deconvolved with a 2D Gaussian PSF model. An exemplary demonstration of the flexibility of SPITFIR(e) is given by the experiments performed with a lattice light sheet microscope^30^ (LLSM) which allows to acquire 3D images for several hours with limited photobleaching. In LLSM imaging, an illumination angle makes the raw image stack skewed, post-processing is required to create a 3D “deskewed” stack^30^. Consequently, deconvolving 3D LLSM images is not straightforward as the PSF is not well characterized and the deskew operation affects the noise statistics. We then design the following SPITFIR(e) deconvolution pipeline according to the flowchart illustrated in Fig. 1. We hypothesized here that chaining the denoising and deconvolution steps could provide better restorations results. So, first we denoise the 3D+Time image sequence (4D denoising) with SPITFIR(e) (the PSF function *h* is a Dirac function). Then we deconvolve each 3D stack separately with SPITFIR(e) by using an anisotropic 3D Gaussian PSF model. The settings of the PSF are chosen given the image resolution along the XY and Z axes.

**Figure 2:**
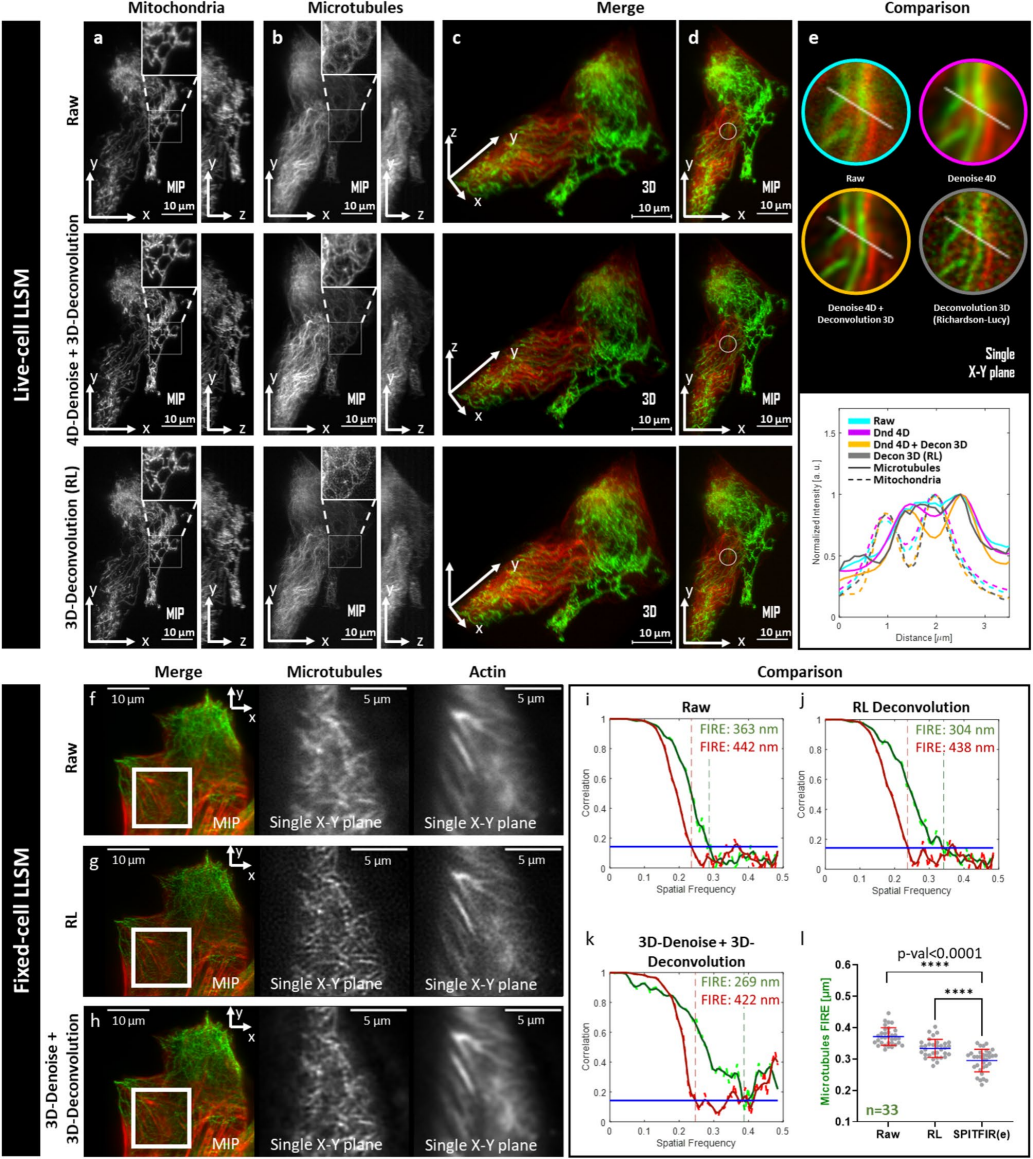
SPITFIR(e) on LLSM images. *Live-cell LLSM. *60 planes 3D volumes of live RPE1 cells double stained with PKMR for Mitochondria (**a** and with Tubulin Tracker Deep Red for Microtubules (**b**) were acquired within 2.2*s* per stack using LLSM. Maximum intensity projection (MIP) of representative raw images labeled for Mitochondria and Microtubules, respectively, before (**a**, **b**; upper part) and after (middle part) SPITFIR(e) 4D denoising + 3D deconvolution (3D Gaussian PSF, $\sigma _{xy} =1.5$ pixels and $\sigma _{z} =1.0$ pixel) and after (lower part) 3D deconvolution (Richardson-Lucy (RL) algorithm with a 3D Gaussian PSF, $\sigma _{xy} =1.5$ pixels and $\sigma _{z} =1.0$; 10 iterations). Insets are zoomed area illustrating SPITFIR(e) improvement in spatial resolution and SNR as compared to RL processing. 3D angular views and MIP of composite images (red for Microtubules; green for Mitochondria) before (**c**, **d**; upper part) and after SPITFIR(e) (middle part) or RL (lower part). Comparative illustrations of 4D denoising SPITFIR(e), 4D denoising+3D deconvolution SPITFIR(e) and RL 3D deconvolution image improvement shown on a single X-Y plane (**e**) zoomed from the ring insets indicated in the three images in (**d**). Raw image is indicated as a blue lined circle (**e**; upper part). 4D denoised image is similarly indicated in magenta, while the 4D denoised + 3D deconvolved image is in yellow, and 3D RL deconvolution in gray. Intensity line profiles (**e**; lower part) were measured as indicated in the 4 circles (**e**; upper part) and plotted (**e**; lower part) for each processing step and both Mitochondria and Microtubules staining. Scale bars are indicated in the bottom right corners ($10 \,\mu \hbox {m}$). 3D+time series related to Raw and 4D denoised + 3D deconvolved SPITFIR(e) data are shown as S1 Video and S2 Video. *Fixed-cell* *LLSM*. RPE1 cells labeled with SiR-Actin were fixed before immunostaining for α-tubulin (**f**–**h**). 60 planes 3D volumes were acquired withing 2 s per stack using LLSM. Representative merged MIP images of actin filaments (red) and microtubules (green) staining for raw images, before (**f**), after Richardson-Lucy (RL) deconvolution (**g**, 3D Gaussian PSF, σ_xy_ = 1.5 pixels and, σ_z_ = 1.0 pixel and 10 iterations) and after SPITFIR(e) 3D denoising and 3D deconvolution (**h**, 3D Gaussian PSF, σ_xy_ = 1.5 pixels and   σ_z _ = 1.0 pixel). Zoomed area of a single X-Y plane (**f**–**h**, middle and right images; scale bars = 5 μm) was extracted from the insets (as indicated in **f**–** h**; left images) for qualitative comparison between SPITFIR(e) and RL. Fourier Image Resolution (FIRE) was estimated using Fourier Ring Correlation Plugin [32] for multiples single X-Y planes before (**i**), after RL 3D deconvolution (**j**) and after SPITFIR(e) 3D denoising + 3D deconvolution (**k**). SPITFIR(e) improvement was estimated separately for 33 X-Y single planes of microtubules from 6 RPE1 cells acquired using LLSM. Statistics analysis is represented in (**l**). It indicates the improvement of average FIRE (unpaired two-sample *t*-test).

We first illustrate the efficiency of the “denoise-before-deconvolve” approach using microtubules and mitochondria internal membrane double labeling (Fig. 2, Live-cell LLSM). Live imaging mitochondria and their interactions with other intracellular components at high spatial resolution in 3D and high temporal frequency, is known to be challenging. Beyond the usual optical limits, labeled mitochondria are very sensitive to light^31^, which could induce artefactual fluorescence signals such as exaltation, probes dissociation and finally swelling and fragmentation of mitochondria and cell death. This may impair accurate 3D+Time observation of their behaviors in normal and stress conditions. For these reasons, imaging mitochondria dynamics and structural features will serve as one of the biological thread in the following parts of the manuscript.

We imaged live RPE1 cells in full volume labeled for mitochondria (Fig. 2 (a); Raw images, upper part) and microtubules (Fig. 2(b); Raw images, upper part). We here linked 3D LLSM at low illumination regimes to efficiently preserve fluorescence from photobleaching and consequently from phototoxic effects, with 4D denoising and 3D image deconvolution by using SPITFIR(e) (Fig. 2 (a, b); middle images) and a comparison with 3D deconvolution using Richardson-Lucy (RL-Deconvolution) approach (Fig. 2 (a, b); bottom images). Results are shown as single labeling (Fig. 2(a, b) and as merged images (Fig. 2(c, d), with different angles of view.

A reconstituted comparison of the successive steps of the SPITFIR(e) workflow, 4D denoised followed by 3D deconvolution (see Supplementary Fig. S1) and 3D RL deconvolution is shown in Fig. 2(e). For the sake of visual clarity, only one single plane of the zoomed area of a stack (as indicated in Fig. 2(d; upper, middle and bottom images) is used, and shown as zoomed insets in Fig. 2(e; upper part) from raw image to full SPITFIR(e) processing and compared to 3D RL deconvolution). More visual representations can be appreciated in supplementary Videos S1 and S2, in 3D+Time along both XY and Z axes. Interestingly, effect of chaining denoising and deconvolution with SPITFIR(e) is particularly visible on the microtubule staining which efficiency is low, while improvement in resolution is more obvious on mitochondria (compare insets of zoomed area between Fig. 2(b) upper and middle images and between Fig. 2(a) upper and middle images). As expected, SPITFIR(e) does not outperform RL improvement in resolution for high SNR images (green for mitochondria), while clearly improving both image quality and resolution for low SNR (red, microtubules). Line intensity plots through the center of the fluorescent filamentous structures (Fig. 2(e); upper part) demonstrate the recovery of the signal from the noise (Fig. 2(e); bottom part). For quantitative measurements of the performance of SPITFIR(e) vs RL using LLSM images we double labeled fixed cells for two filamentous intracellular structures, the microtubules (green) and the actin filaments (red). Merged images and zoomed extracts are shown on Fig. 2(f, g, h, Fixed-cell LLSM). We computed the Fourier Image Resolution (FIRE) using Fourier Ring Correlation Plugin^32^ for multiple single X–Y planes before (Fig. 2(i)), after RL 3D deconvolution (Fig. 2(j)) and after SPITFIR(e) 3D denoising + 3D deconvolution (Fig. 2(k)). For both labeled structures SPITFIR(e) using 3D denoising before 3D deconvolution improves the resolution as compared to RL deconvolution, again with a more pronounced effect in the lowest SNR fluorescent channel (compare green and red curves in Fig. 2(i, j, k)). This clear improvement in the lowest SNR fluorescent channel is statistically relevant (33 X–Y planes, from 6 different cells) as confirmed by the results shown in Fig. 2(l).

**Figure 3:**
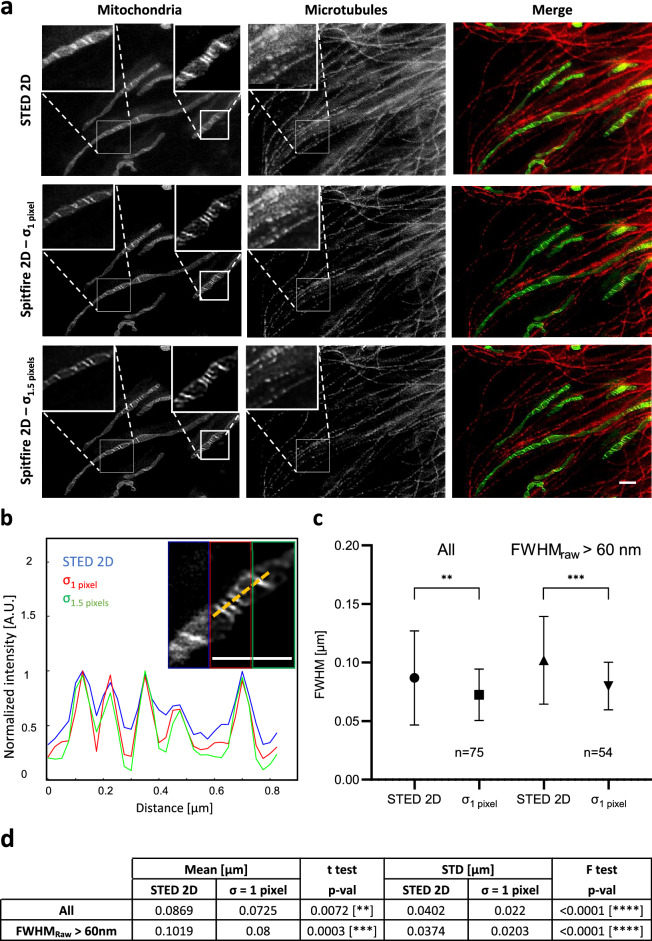
SPITFIR(e) increases the signal-to-noise of low-light images in STED imaging. Live RPE1 cells double stained with PKMO (mitochondria) and Tubulin Tracker Deep Red (microtubules) were imaged by STED nanoscopy before and after being processed by SPITFIR(e) (**a**). Partial overview of 2D STED raw data of RPE1 cells for mitochondria, microtubules and their superimposed images are shown from left to right (**a**, upper panel). 2D STED images were improved by SPITFIR(e) using two different 2D Gaussian model $\sigma _{xy} =1.0$ pixel (**a**, middle panel) and $\sigma _{xy} =1.5$ pixels (**a**; lower panel). Insets (1 and 2) show zoomed area where SPITFIR(e) image quality improvement is illustrated by comparison of two processing conditions (middle and lower panel) with the same area in the raw STED image. Inset(**b**) shows a magnified composite image where the 2D STED raw part is indicated as a blue lined rectangle and 2D denoised + 2D deconvolved parts are indicated in red ($\sigma _{xy} =1.0$ pixel) and green ($\sigma _{xy} =1.5$ pixels). (**b**) Normalized intensity line profiles were measured in the insets (**b**) for cristae regions. Yellow line indicated in the inset (**b**) serve to identify fluorescence profiles. Pixel size is equal to 25 nm. Scale bars: $1 \,\mu \hbox {m}$ in (**a**) and (**b**). Line profiles of individual cristae (n=75, from 8 different acquisitions) were fitted using a Gaussian model. Full width half maximum (FWHM) was estimated on raw STED and 2D denoised + 2D deconvolved with $\sigma _{xy} = 1.0$ pixel (**c**). SPITFIR(e) improvement is shown separately for all the line profiles analyzed and the line profiles from bigger cristae ($\text {FWHM}_{raw} = 60 \,\hbox {nm}$), indication of lower SNR. Statistics analysis is including in (**d**). It indicates the improvement of average resolution (unpaired two-sample *t*-test) and variance of cristae resolution (*F*-test).

With SPITFIR(e), we have also improved the resolution limits of current STED microscopy. STED microscopy provides subdiffraction resolution while preserving useful aspects of fluorescence microscopy, such as optical sectioning, and molecular specificity and sensitivity. The PSF shape depends on several factors such as optics, laser power, fluorescence response of the specimen, and thermal drift which can add deformation by sheering. STED nanoscopy is one of the very few light microscopy techniques^33,34^ allowing to solve mitochondria cristae organization. STED microscopy provides subdiffraction resolution while preserving useful aspects of fluorescence microscopy, such as optical sectioning, and molecular specificity and sensitivity. The PSF shape depends on several factors such as optics, laser power, fluorescence response of the specimen, and thermal drift which can add deformation by sheering. In Fig. 3, we deconvolved with SPITFIR(e) two 2D images depicting live mitochondria and their alignment on microtubules, by applying SPITFIR(e) 2D with a 2D Gaussian PSF model and two different bandwidths $\sigma _{xy}$ of size 1.5 pixels and 1.0 pixel, respectively (Fig. 3(a)). Importantly, we tested and obtained very similar results with a Lorentzian 2D PSF with such bandwidth values (not shown as the differences are nearly indistinguishable), mainly because the PSF sizes are small in these experiments corresponding to few pixels when implemented in the discrete setting. We selected two regions of interest (ROI) depicting mitochondria cristae to better appreciate the restoration results in (Fig. 3(a, b)). Normalized fluorescence intensity line profiles, positioned at a distinct cristae region of mitochondria (Fig. 3(b) and Supplementary Fig. S2) show the improvement of SPITFIR(e) in terms of SNR and image quality, with no visual artifact and noise amplification. Two simulated PSFs sizes were proposed to evaluate the capacity of SPITFIR(e) to improve the image resolution. The first PSF ($\sigma _{xy}=1.0$ pixel) was similar than the expected experimental PSF of our microscope and the second PSF ($\sigma _{xy}=1.5$ pixels) corresponds to the situation where there is an issue of defocusing. Clearly in both cases we observe an image improvement of the two individual cristae line profiles. The fitting of a Gaussian model to intensity profiles ($n=75$, from 8 different acquisitions) allowed us to estimate the resolution at FWHM of raw and restored images (Fig. 3(b, c)). The obtained resolution is statistically improved from direct STED imaging when SPITFIR(e) is applied (with a smaller variance), as shown in (Fig. 3(d), and correspond to twice the reported results reported in transmitted electron microscopy^35^. Finally, we compared the resolution achieved by SPITFIR(e) and RL as shown in Supplementary Fig. S2. As examined by intensity profiles, resolutions reached by both algorithms are very similar. However, deconvolution artifacts are strongly reduced using SPITFIR(e) (Supplementary Fig. S2(c)).

### SPITFIR(e) reliably restores weak fluorescent signals under extreme low-light conditions

Additional demonstrations of the ability of SPITFIR(e) to retrieve the high spatial-frequency information under extreme light conditions in 3D multifocus microscopy and spinning-disk confocal microscopy, are presented below. We focus on the performance limits of 3D deconvolution knowing that it is possible to improve the results by denoising the images beforehand as described above.

**Figure 4:**
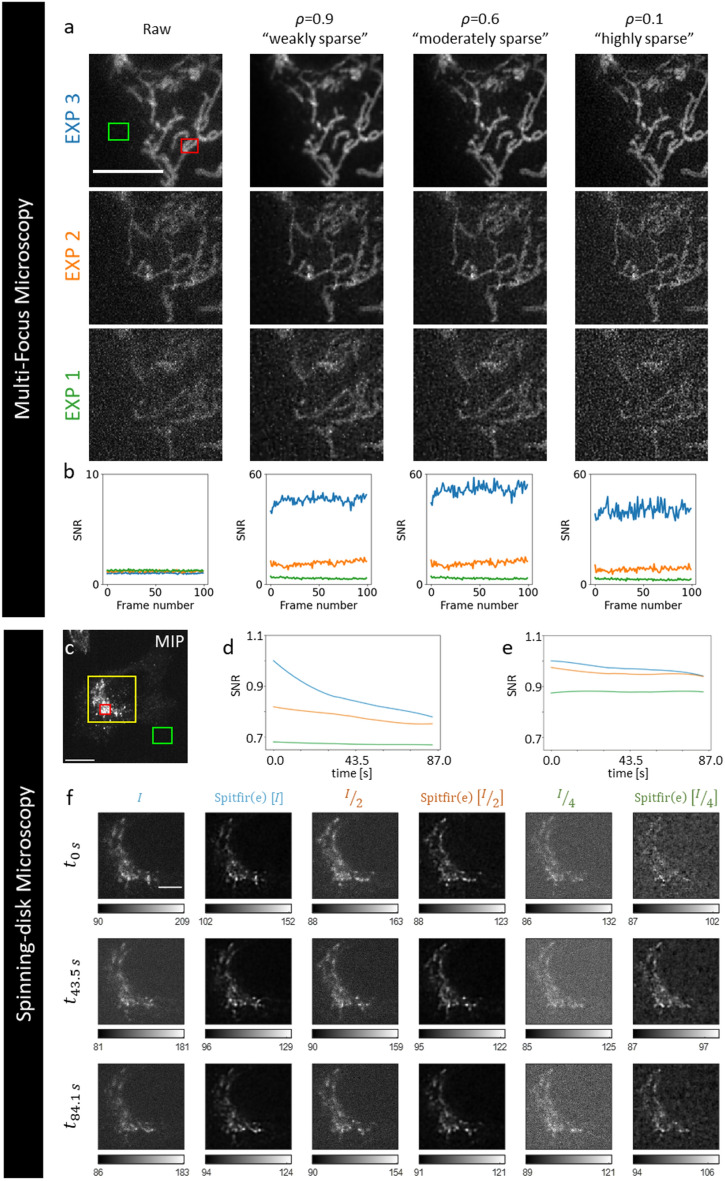
SPITFIR(e) image reconstruction applied to wide-field multifocus and spinning-disk confocal microscopy with different amounts of sparsity, exposure and illumination values. *Multifocus microscopy.* Data are temporal series (100 time points) of 3D stacks composed of nine planes each depicting mitochondria in U2OS cells transfected with TOM20 (translocase of outer mitochondrial membrane) fused to GFP (GFP-TOM20). Exposure time: 50 ms. The sample is imaged with three different doses of illumination light. MFM allows simultaneous acquisition of a 3D stack of nine images equally spaced with (dz = 330 nm) focal 2D images (pixel size = 120 nm, see also Supplementary Figure S3). (**a**) The image grid display the deconvolution results on the 6th plane for successive increasing laser power values (EXP 1, 2 and 3). The 3D stacks have been deconvolved with 3D Gaussian PSF model (σ_xy_ = 1.5 pixels and σ_z_ = 1.5 pixels) and with three different levels of sparsity (“weak”, “moderate”, and “high”). (**b**) The plots are SNR values calculated for the 6th plane at each time-point as follows: SNR = Power(signal) / Power(background) = |Ω(x_S_)|^-1^ ∑$_{\rm x\in\Omega (\rm x_{S})}$I(x)^2^ / |Ω(x_B_)| ^-1^ ∑$_{\rm x\in\Omega (\rm x_{B})}$ I(x)^2^, where Ω(x_S_) (with size |Ω(x_S_)|) and Ω(x_B_) (with size |Ω(x_B_)|) are two ROIs centered at pixels x_S_ (red ROI) and x_B_ (green ROI), respectively. Scale bar: 10μm. *Spinning-disk microscopy.* Data are temporal series (30 time points) of 3D stacks composed 14 planes each depicting CD-M6PR-eGFP Hela cells. Exposure time: 20 ms, Δt between two stacks: 2.9 s. The sample is imaged 3 times with different doses of illumination light. (**c**) The figure depicts a MIP (Maximum Intensity Projection) of the 3D stack at t_0s_, acquired with a relatively high laser power dose (intensity *I*) (Scale bar = 10 μm). The plots represents SNR values measured overtime on the single 6th plane (time in seconds) for three time series acquired with *I*/4, *I*/2, *I* of laser illumination, successively before (**d**) and after (**e**) applying SPITFIR(e). The SNR metric is defined as above with two ROIs centered at pixels x_S_ (red ROI in **c**) and x_B_ (green ROI in **c**), respectively. The figure (**f**) shows a zoomed area (yellow in **c**) of the 6th plane at time t_0s_, t_43.5s_ and t_84.1s_ extracted from the full movies acquired with high dose, half and one fourth dose of illumination light before (*I*, *I*/2 *I*/4) and after applying 4D denoising + 3D deconvolution SPITFIR(e) (Spitfir(e) [*I*], Spitfir(e) [*I*/2]) and Spitfir(e)[*I*/4]) to the three 4D volumes, with a Gibson-Lanni PSF model truncated to 3 planes around the center of the PSF. Intensities “min” and “max” are indicated as grey level bars, under each thumbnail images. Image contrast was adapted for visualization purposes. Scale bar: 4.5μm.

First, we tested SPITFIR(e) to visualize the 3D dynamics of mitochondria in living cells under extreme low-light conditions with 3D multifocus microscopy (MFM; see Supplementary Fig. S3) for different exposure values by reducing the excitation light dose (see Fig. 4(a)). In this experiment, each 3D stack was restored with a 3D Gaussian PSF model. The results on 2D plane (6th plane in Fig. 4(a)) are shown in Fig. 4 for three different illumination values using the same sample. We considered three different levels of sparsity: “highly”, “moderately”, and “weakly” sparse. In Fig. 4(b), we display the increasing of SNR values for 100 time points computed from two ROIs selected in the foreground (red rectangle) and background (green rectangle) in Fig. 4(a, top left image). The results computed for the three different levels of sparsity demonstrate the ability of SPITFIR(e) to reduce noise while enhancing small structures and removing background. Overall, the most satisfying results with SPITFIR(e) are obtained with “moderate” sparsity, (Fig. 4(a) third column, and Supplementary Fig. S3(c) vs. (d)).

We further evaluated the flexibility of SPITFIR(e) on spinning-disk confocal microscopy images. Multi-pinholes spinning-disk microscopes are compatible with acquisition regimes allowing high speed live cell imaging. Nevertheless, notably because of photobleaching effects, time exposure must be reduced in order to capture very fast intracellular events, such as membrane trafficking. We acquired images with different excitation light doses in order to increase the frame rate and capture fast movements while reducing photobleaching and photo-toxicity effects. To assess the overall sensitivity of SPITFIR(e) cells expressing Calcium Dependent Mannose 6 phosphate Receptor-eGFP (CD-M6PR-eGFP) were imaged at variable excitation light levels, reducing light intensity by a factor of 2 and 4, as shown in Fig. 4(d). The three time-lapse temporal series display bleaching curves which correlates with the excitation light intensity levels and SNR values (calculated from Fig. 4(c), and following the equation displayed in figure legend). As expected, when we reduce by half or one fourth the laser power (raw data denoted *I*/2 and *I*/4 in Fig. 4(f) and orange and green curves in Fig. 4(d, e), the bleaching effect is reduced and the SNR is lower, as compared to data with illumination at *I*. We applied the proposed SPITFIR(e) restoration confocal pipeline on the three 3D+Time series. As shown in Fig. 4(e), SPITFIR(e) restore the SNR for low (*I*/2 orange curve and *I*/4 green curve) illumination conditions, while removing the time dependent photobleaching effect for high (*I*, blue curve) or intermediate (*I*/2 orange curve) laser power illumination. Beyond these quantitative measurements, we also explore the SPITFIR(e) image quality rendering in these experiments. Figure 4 (f), display 3D+Time images *I*, *I*/2, *I*/4 for three time points of the corresponding temporal series illustrating broadly the SPITFIR(e) restoration pipeline performance. While image quality is reduced for the lowest illumination intensity (*I*/4), the restored images are good enough for spot detection. More importantly, SPITFIR(e) preserves SNR and thus restore image quality even at high frame rate and high laser excitation (compare *I* and Spitfir(e)[*I*] at $t_{84.1 \textrm{s}}$ in Fig. 4(f)). As other experiments complementarily depicting different intracellular structural features, Supplementary Fig. S4 shows the maximum intensity projection along the Z-axis of a single stack depicting Rab5 proteins coupled to Green Fluorescence Protein (Rab5-eGFP), labelling early endosomes, for which the original images are noisy and contains diffraction limited spots against a dark background, while Supplementary Fig. S5 shows the maximum intensity projection along the Z-axis of a stack depicting the thin filamentous structures of polymerized actin (mCherry-LifeAct). The latter image is apparently not as sparse as the Rab5-image. These two examples are challenging because the PSF is known approximately and the image contents are very different. The 3D PSF is assumed to be approximated by a 3D Gaussian function. In each figure, we reported the results obtained with four SPITFIR(e) strategies (3D deconvolution, PbyP deconvolution, 3D denoising + 3D deconvolution, 3D denoising + PbyP deconvolution) and different levels of sparsity. Applying denoising beforehand enables here to better enhance weak fluorescent signals while removing blur and background. From Supplementary Figs. S4 and S5, it visually best results on the Rab5-eGFP and LifeActin-mCherry images are obtained when the sparsity is “high” and “moderate”, respectively.

We conclude that even at low photon counts and low SNR, SPITFIR(e) helps to preserve sample integrity, while allowing to extract exploitable data for quantitative analysis.

### SPITFIR(e) quantitatively outperforms state-of-the-art algorithms

In fluorescence microscopy, restoration algorithms were dedicated to remove Poisson-Gaussian noise originating from the low-photons regimes (Poisson noise) and dark current induced by the electronic imaging detectors (Gaussian noise). In Methods (and Appendix), we formally demonstrate that a quadratic fidelity term combined with any regularization term is optimal to handle Poisson-Gaussian noise. This new result allows us to apply SPITFIR(e) on images acquired with low light doses (Fig. 4) and to quantitatively compare state-of-the-art deconvolution algorithms mainly developed for Gaussian noise removal, and applied here to fluorescence microscopy images.

First, we fairly benchmarked the performance of a large collection of competitive deconvolution algorithms given in Table 2, including the popular deconvolution methods such as RL, iterative constrained Tikhonov-Miller (ICTM), Gold-Meinel (GM))^36^. As the implementation in 3D of several deconvolution algorithms is not always possible, we conducted experiments on four 2D images shown in Table 1. The four ground-truth images were normalized in the range [0, 1] and further blurred by considering a Gaussian point spread function (PSF) with different standard deviation values $\sigma _{xy}$. A Gaussian noise with zero mean and standard deviation $\tau$ was also added to these images in order to generate observed noisy and blurry data. Note that, in the case of RL algorithm, which is originally designed to deal with Poisson noise, the degraded images are re-scaled to the original dynamics of the underlying reference images. The RL deconvolution results are then re-normalized for a fair comparison with those obtained by the other methods. Moreover, before applying GM, the noisy images are smoothed with a Gaussian filter as pre-processing step because this algorithm assumes that the noise is negligible. In these experiments, we consider three different PSF sizes which correspond to $\sigma _{xy} \in \lbrace 1, 1.25, 1.5 \rbrace$ and three distinct noise levels $\tau \in \lbrace 0.01, 0.02, 0.04 \rbrace$ (intensities are in the range [0, 1]). In Table 1, we reported the best possible PSNR values for each method (with optimal parameter adjustment) and the true PSF size $\sigma _{xy}$ and noise standard deviation $\tau$. In this experiment, RL and GM algorithm were often ranked at the end of the benchmark, while SPITFIR(e) usually provided the best PSNR values.

**Figure 5:**
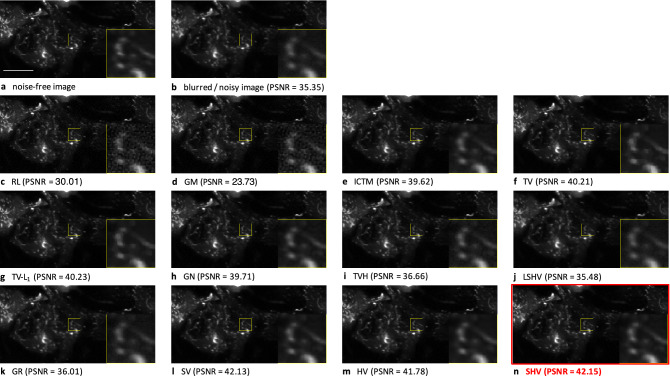
Deconvolution of a confocal microscopy image with different methods. (**a**) The original 512 $\times$ 256 pixels image (Image #1) (source: CNRS UMR144 Institut Curie) is a single 2D plane extracted from a 77 time frame series depicting Rab11a-mCherry protein in M10 cells, was previously acquired in line scanning confocal microscopy. Scale bar: 10 $\mu$m. (**b**) Artificially corrupted image by a Gaussian PSF $\sigma _{xy} = 1.0$ pixel and Gaussian white noise with standard deviation $\tau = 0.01$ (**b**). (**c**) Image deconvolved with the Richardson-Lucy (RL)^3^ algorithm. (**d**) Image deconvolved with the Gold-Meinel (GM)^39^ algorithm. (**e**) Image deconvolved with the iterative constrained Tikhonov-Miller (ICTM)^9^ algorithm. (**f**) Image deconvolved with the Total Variation (TV)^40^ regularizer. (**g**) Image deconvolved with the Total-Variation-L1 (TV-L1)^21^ . (**h**) Image deconvolved with the GraphNet (GN)^17^ regularizer. (**i**) Image deconvolved with the Total Variation-Huber (TVH)^41^ regularizer. (**j**) Image deconvolved with the Log Sparse Hessian Variation (LSHV)^16^ regularizer. (**k**) Image deconvolved with the Good’s roughness (GR)^42^ regularizer. (**l**) Image deconvolved with the Sparse Variation (SV)^20^ regularizer. (**m**) Image deconvolved with the Hessian Variation (HV)^14,15^ regularizer. (**n**) Image deconvolved with the Sparse Hessian Variation (SHV) (our SPITFIR(e) method) regularizer (black box). Zoom-in views are displayed in yellow windows for comparison in details.

In terms of visual assessment, we display the noise-free versus artificially blurred and noisy images (Image #1) depicting small fluorescent vesicles acquired with classical laser scanning confocal microscopy in Fig. 5(**a**) and Fig. 5(b)), respectively. In this example, the original image was corrupted by a Gaussian PSF ($\sigma _{x,y} = 1$ pixel) and an additive white Gaussian noise with standard deviation $\tau = 0.01$ (corresponding to a value of PSNR = 35.35 dB). In Fig. 5(c) and Fig. 5(d), we can notice noise amplification with RL and GM, respectively, resulting in the apparition of undesired high-intensity pixels (i.e., the “night sky” effect) and unrealistic reconstructed structures since the high-frequency components were not correctly restored. ICTM produced deconvolution results without noise amplification artifacts but tends to over-smooth image details due to the quadratic nature of Tikhonov-Miller penalty (see Fig. 5(e)), The GN regularizer, which also incorporates Tikhonov penalty, provides indeed a similar deconvolved image with blurred details (see Fig. 5(h)) while comparing to the ICTM solution. In contrast to these over-smoothed results, TV (see Fig. 5(f)), TV-$L_1$ (see Fig. 5(g)) and SV (see Fig. 5(l)) regularizers generate artificial sharper edges (stair-casing effects), respectively. HV generates visually more pleasant deconvolution results (see Fig. 5(m)). Regarding smooth-approximation based regularizations, LSHV (see Fig. 5(j)) provides results with restored details which are slightly sharper than those obtained with ICTM (see Fig. 5(e)), but noise is not sufficiently removed. In the comparison with TVH (Fig. 5(i)) and LSHV (Fig. 5(**j**)), the smooth version of GR (Fig. 5(k)) yields visual similar deconvolution results. However, GR suffer from severe “white pixel” artifacts in the background region. These “white pixels” were discarded beforehand to fairly compute the PSNR values. Finally, SPITFIR(e) (SHV) avoids over-smoothing and over-sharpening effect while preserving details and removing noise in the background (see Fig. 5(n)). These quantitative results were confirmed on 3D microscopy images by considering additional performance criteria.

**Table:** 

Image #1 (512×256)(CM)	Image #2 (227×238)(TIRFM)	Image #3 (256×283)(TIRFM)	Image #4 (1054×1028)(SIM)

**Table 1:** PSNR scores of deconvolution methods applied to four 2D images (confocal (CM), TIRFM, SIM). The best scores ($\pm 0.05\; {\textrm{dB}}$) are in bold style (including when the error is lower than 0.05 dB). Images #1-#3 depict fluorescently tagged proteins (Rab11-mCherry, Langerin-YFP, TfR-pHluorin (Transferin-receptor)) corresponding to small bright spots over a dark background, observed in spinning-disk confocal microscopy (CM) (Image #1, courtesy of PICT-IBiSA imaging platform) and total internal reflection fluorescence microscopy (TIRFM) (Images #2 and #3) respectively, previously acquired^37,38^. Image #4 depicts microtubules observed in Structured Illumination Microscopy (SIM) with high resolution (up to 100 nm). This image (CIL 36147) is taken from the Cell Image Library (CIL) (https://www.cellimagelibrary.org). The images were deconvolved with the following methods: Richardson-Lucy (RL)^3^ algorithm, Gold-Meinel (GM)^39^ algorithm, iterative constrained Tikhonov-Miller (ICTM)^9^ algorithm, Total Variation (TV)^40^ regularizer, Total-Variation-L1 (TV-L1)^21^, GraphNet (GN)^17^ regularizer, Total Variation-Huber (TVH)^41^ regularizer, Log Sparse Hessian Variation (LSHV)^16^ regularizer, Good’s roughness (GR)^42^ regularizer, Sparse Variation (SV)^20^ regularizer, Hessian Variation (HV)^14,15^ regularizer, and Sparse Hessian Variation (SHV) (our SPITFIR(e) method) regularizer. The PSNR values correspond to optimal settings of parameters for all competing methods, including SPITFIR(e). We deconvolved the four images several times with different parameters to objectively supply the best PSNR value as possible, for all methods. The PSNR values are averaged values conventionally computed from 3 realizations (standard deviation less than 0.05 db).

		RL	GM	ICTM	TV	TVL1	GN	TVH	LSHV	GR	SV	HV	SHV (SPITFIR(e))
Image #1 (CM)	$\sigma _{xy} = 1.0$												
	$\tau = 0.01$	30.01	23.73	39.62	40.21	40.23	39.71	36.66	35.48	36.01	**42.13**	41.78	**42.15**
	$\tau = 0.02$	25.12	19.89	37.04	38.04	38.06	37.10	32.98	32.75	32.93	39.45	39.34	**39.55**
	$\tau = 0.04$	20.52	15.89	34.40	35.76	35.76	34.43	28.56	28.52	28.47	**36.68**	36.43	36.61
	$$\sigma _{xy} = 1.25$$												
	$\tau = 0.01$	31.02	28.92	38.52	38.88	38.86	38.61	35.88	33.96	34.55	**40.84**	40.42	**40.87**
	$\tau = 0.02$	25.95	24.11	36.35	36.81	36.80	36.41	32.56	31.88	32.20	**38.65**	38.28	38.57
	$\tau = 0.04$	21.02	19.36	33.90	34.87	34.90	33.99	28.42	28.17	28.27	**36.09**	35.80	35.96
	$$\sigma _{xy} = 1.5$$												
	$\tau = 0.01$	31.85	32.89	37.50	37.95	37.89	37.61	35.20	32.70	33.28	**39.87**	39.12	39.61
	$\tau = 0.02$	26.86	27.92	35.51	36.04	36.03	35.57	32.15	31.05	31.44	**37.78**	37.19	37.69
	$\tau = 0.04$	21.67	22.65	33.25	33.86	33.86	33.34	28.23	27.73	27.84	**35.34**	34.83	35.08
Image #2 (TIRFM)	$\sigma _{xy} = 1.0$												
	$\tau = 0.01$	27.61	10.66	31.08	30.92	**31.29**	31.00	30.73	29.79	30.73	30.92	31.01	31.01
	$\tau = 0.02$	22.81	29.64	29.79	29.77	**30.01**	29.69	28.72	28.56	28.65	29.83	29.89	**30.01**
	$\tau = 0.04$	17.93	27.50	28.27	28.40	28.43	28.21	25.75	25.75	25.76	28.60	28.63	**28.78**
	$$\sigma _{xy} = 1.25$$												
	$\tau = 0.01$	28.26	17.07	29.96	29.78	**30.13**	30.00	29.73	28.42	29.75	30.01	29.87	30.01
	$\tau = 0.02$	23.94	15.37	28.99	28.94	**29.23**	28.95	27.94	27.53	27.92	29.17	29.02	29.16
	$\tau = 0.04$	18.98	12.13	27.64	27.68	27.77	27.58	25.22	25.21	25.22	27.97	27.85	**28.09**
	$$\sigma _{xy} = 1.5$$												
	$\tau = 0.01$	28.46	23.29	28.97	28.80	28.91	27.38	28.93	**29.09**	29.07	**29.09**	28.90	**29.08**
	$\tau = 0.02$	24.76	20.61	28.17	28.10	28.38	28.19	27.35	26.72	27.29	**28.44**	28.04	28.29
	$\tau = 0.04$	19.94	16.54	26.98	26.97	27.10	26.91	24.81	24.74	24.78	**27.34**	27.08	**27.35**
Image #3 (TIRFM)	$\sigma _{xy} = 1.0$												
	$\tau = 0.01$	28.19	13.56	33.30	33.05	**33.40**	33.22	32.64	32.16	32.68	33.07	33.27	33.27
	$\tau = 0.02$	23.21	11.75	**31.96**	31.50	31.74	31.89	30.27	30.26	30.26	31.49	31.83	31.82
	$\tau = 0.04$	18.26	28.65	30.40	30.20	30.23	30.34	26.86	26.85	26.87	30.20	**30.50**	**30.51**
	$$\sigma _{xy} = 1.25$$												
	$\tau = 0.01$	29.11	19.47	32.42	32.14	**32.49**	32.28	31.86	30.99	31.83	32.22	32.34	32.34
	$\tau = 0.02$	24.31	16.88	**31.24**	30.82	31.19	31.15	29.67	29.51	29.63	30.81	31.11	31.14
	$\tau = 0.04$	19.28	12.99	**29.76**	29.42	29.51	29.68	26.47	26.46	26.46	29.60	29.68	**29.76**
	$$\sigma _{xy} = 1.5$$												
	$\tau = 0.01$	29.69	25.03	**31.51**	31.36	31.62	31.39	31.13	29.98	31.12	31.36	**31.48**	**31.48**
	$\tau = 0.02$	25.31	21.46	**30.60**	30.18	30.55	30.49	29.15	28.80	29.14	30.33	30.42	30.40
	$\tau = 0.04$	20.23	16.86	**29.26**	28.86	28.98	29.19	26.17	26.15	26.08	**29.21**	29.05	**29.24**
Image #4 (SIM)	$\sigma _{xy} = 1.0$												
	$\tau = 0.01$	31.24	21.49	36.57	35.87	35.93	37.06	36.19	33.74	36.14	37.89	37.62	**38.21**
	$\tau = 0.02$	25.89	19.17	33.95	33.76	33.77	34.42	32.60	31.89	32.60	35.19	34.99	**35.54**
	$\tau = 0.04$	20.80	15.66	31.33	31.41	31.44	31.79	28.41	28.31	27.66	32.48	32.31	**32.70**
	$$\sigma _{xy} = 1.25$$												
	$\tau = 0.01$	32.13	28.63	34.79	34.55	34.56	35.35	34.58	31.46	34.53	36.08	35.66	**36.29**
	$\tau = 0.02$	26.82	24.75	32.55	32.36	32.40	33.09	31.67	30.33	31.64	33.79	33.49	**34.07**
	$\tau = 0.04$	21.45	19.94	30.29	30.29	30.35	30.67	28.03	27.64	27.35	31.41	31.13	**31.54**
	$$\sigma _{xy} = 1.5$$												
	$\tau = 0.01$	32.34	33.04	33.14	33.36	33.40	33.70	33.08	29.82	33.09	34.47	34.00	**34.66**
	$\tau = 0.02$	27.55	28.78	31.27	31.19	31.25	31.80	30.67	29.03	30.66	32.48	32.08	**32.64**
	$\tau = 0.04$	22.15	23.57	29.36	29.37	29.46	29.88	27.60	26.94	27.04	30.44	30.08	**30.54**

We next conducted experiments on 3D confocal images and compared the results of SPITFIR(e) to those produced by CARE^43^, a supervised deep-learning-based deconvolution method (Supplementary Fig. S6). As CARE is learned from a training set of dedicated biological structures, we evaluate the algorithms on the stack provided by the authors^43^ and depicting the envelopes of nuclei stained with GFP-LAP2b (developing eye of zebrafish (Daniorerio) embryos. For the sake of visual assessment, Supplementary Fig.  S6 only shows the restoration of the 5th plane of an anisotropic 3D stack composed 18 images^43^. As expected, the reconstruction quality is visibly superior with CARE. Nevertheless SPITFIR(e) with 3D Gaussian PSF ($\sigma _{xy} = 2.0$ pixels, $\sigma _z = 0.5$ pixel) and Gibson-Lanni model^44^ (truncated to 3 planes around the center of the PSF) provided satisfactory results. The main differences are visible on small filaments better enhanced with CARE. The results can be visually improved with SPITFIR(e) if the volume is denoised beforehand and deconvolved with the PbyP strategy (Supplementary Fig.  S6(a; right)).

**Figure 6:**
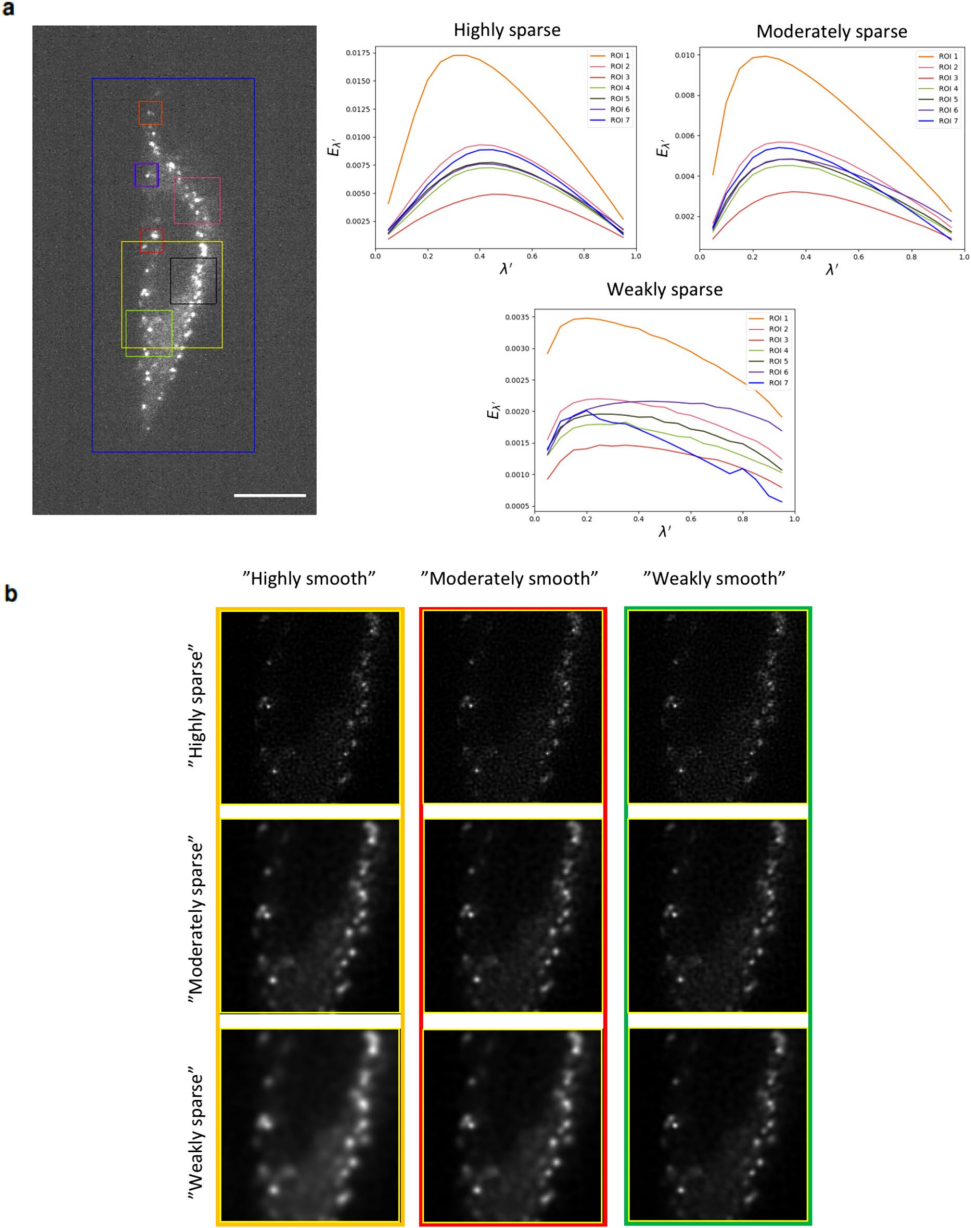
Selection of regularization and sparsity parameters. (**a**) Display of square ROIs with variable sizes superimposed on single X-Y slide of 3D LLSM image depicting $\sigma$ subunit-eGFP of the AP2 complex in Hela cells (left). Scale bar: 10 $\mu$m. Illustrations of energies $E_{\lambda ^\prime }(f, u)$ with respect to $\lambda ^\prime \in ]0, 1[$ for different ROIs, and three levels (“weak”, “moderate”, “high”) of sparsity. The minimax value $\lambda ^{\prime ^*}$ is in the interval [0.2, 0.4] according to the sparsity level in all ROIs, suggesting that the maximum is invariant to scale (right). (**b**) Display of the $3 \times 3$ matrix associated to the yellow ROI (see (**a**), left) whose the rows and columns represent smoothness and sparsity. The default value $\lambda ^\prime = 1/2$ corresponds to matrix cells marked in red. The minimax value $\lambda ^{\prime ^*}$ corresponds matrix cells marked in orange. We also display the “symmetric” solution corresponding to $\lambda ^\prime = 1 - \lambda ^{\prime ^*}$ (matrix cells marked in green).

## Discussion

An important challenge in live fluorescence imaging is to limit photo-damages on the observed sample by reducing the dose of excitation light, while at the same time collecting enough photons to produce informative images for quantitative analysis. To overcome the difficulties of acquisition of 2D+Time and 3D+Time images at low excitation dose and boost the signal corrupted by blur and different sources of noise (shot noise, readout noise), we developed SPITFIR(e), a supermaneuverable and easy-to-use solution able to generate spatially consistent and high-quality fluorescence images. The approach is based on the fast minimization of a convex energy composed of a novel sparse-promoting regularization term and a Poisson-Gaussian-aware quadratic fidelity term. Unlike previous methods which were often tested on a limited number of microscopy modalities^15,16,23^ and need a careful adjustment of regularization parameters to obtain high-quality restoration results, SPITFIR(e) is a nearly automatized method which adapts to a broad range of microscopy techniques (here up to 6), techniques, whatever the detector technology (CCD, sCMOS, PMT, Avalanche photodiodes), and image dimensionality. With this unique algorithm, the practitioner can switch from 4D image denoising to 3D deconvolution, or can chain the two processing tasks to obtain high-quality images. It is only needed to supply the measured PSF or the parameters of the 2D or 3D theoretical PSF (e.g., Gaussian) model, and to specify the level of desired sparsity (“weak”, “moderate”, “high”). A preview of restoration results obtained on a 2D ROI, as illustrated in Fig. 6, is displayed to help the practitioner selecting the amount of sparsity and smoothness. We demonstrated on experimental data that SPITFIR(e) extracted high frequency information and achieved significant improvement in the output quality even with a poor calibrated PSF. Nevertheless, SPITFIR(e) could miss some details in noisy images taken at low excitation light intensity or short exposure time. As the lower light intensities approach the limits of ability of SPITFIR(e) to recover information reliably, a successful strategy consists in chaining denoising (3D or 4D) and deconvolution with a 2D or 3D PSF model. This idea was already presented by the authors^2^. In live experiments, fast acquisition with a high frame rate is generally required to capture fast events^1^, inducing low SNR values. In presence of Poisson shot noise and readout noise, our results demonstrated that the proposed strategy is robust and able to boost signals reliably, and confirms that the quadratic fidelity term is suitable for Poisson-Gaussian noise as theoretically proved (see "Methods").

We have showed that SPITFIR(e) substantially reduces noise and provides better high-quality images than other currently available methods over a wide variety of experimental conditions. In the particular case of LLSM, which is designed already for long lasting fast imaging of 3D large and close to isotropic volumes, SPITFIR(e) shows all its power, since it not only improves resolution and reduces noise, but still allows an increase in the imaging rates by permitting to reduce the illumination dose. SPITFIR(e) also achieved high resolution in sub-diffracted and superresolution microscopy under low-light conditions, without perturbing the signal amplitudes. For two-dimensional live-cell STED imaging, line intensity profiles along mitochondria cristae showed that SPITFIR(e) reached an improved lateral resolution of a factor of 1.3 (see Fig. 3(c, d)). Little is known about the actual dynamics of mitochondrial cristae in multiple physiological processes because of the visualization challenge of cristae structure in living cells. Hessian-SIM modality^33^ or STED nanoscopy^34,45^ are the only approaches so far, able to achieve this goal. While only unique 2D STED images of live mitochondria are shown here, SPITFIR(e) applied to STED images might be of high interest in this context since it enables to restore these fine structures even when STED depletion is incomplete and scanning imaging at very low illumination, making live STED relatively long-term imaging accessible.

More generally, unlike smoothness regularizers, our sparse-promoting regularizer is able to reveal small details in the raw data, while preserving signal peaks and removing background, with no discernible artifact in the final restored images. We also demonstrated that on 2D/3D benchmarks that SPITFIR(e) outperforms existing deconvolution algorithms. Performance has been quantitatively studied in terms of PSNR values on artificially degraded 2D images and in terms of FRC on 3D experimental data using Fourier Image Resolution (FIRE^32^) (Fig. 2).

Finally, we showed that SPITFIR(e) is able to produce comparable results to supervised machine-learning techniques^43^. Unlike content-dependent methods based on deep-learning algorithms requiring many training images, SPITFIR(e) is a method which utilizes sparsity and smoothness as *a priori* knowledge and adapts to any image contents.

In conclusion, our results on experimental data endorse that SPITFIR(e) may be considered as a “swiss-knife”, able to handle different samples imaged with various fluorescence microscopes, adapt to various sources of signal degradation, image contents, and variable signal-to-noise ratios. This algorithm does not require a well calibrated PSF and is easily controlled by choosing three levels of desired sparsity, that is “high”, “moderate”, and “weak”. SPITFIR(e) resolves 3D structures and dynamics of biological components observed at low excitation light intensity and thus holds the promise to push the spatiotemporal resolution limit of sub-diffracted fluorescence microscopy technique.

## Methods

### Variational model for image restoration

An observed 2D/3D image $f: \Omega \subset {{\mathbb {R}}}^d \rightarrow {{\mathbb {R}}}, d=2,3$ is a blurred and noisy version of the underlying true image $u: \Omega \rightarrow {{\mathbb {R}}}$ (i.e., $u \in {{\mathbb {R}}}^\Omega$). A general approach for restoring an image consists in finding the minimizer of an energy functional, i.e.,

1 $$\begin{aligned} {{\hat{u}}} = \arg \min _{u \in {{\mathbb {R}}}^\Omega }\; F(f,u; h, {{\mathcal {T}}}) + \lambda R_\rho (u) \end{aligned}$$

where $F(f,u; h, {{\mathcal {T}}})$ and $R_\rho (u)$ are the data fidelity term and the regularization term, respectively, and $\lambda >0$ and $\rho \in [0, 1]$ are the parameters controlling the amount of smoothness and sparsity in the image *u*. The fidelity data term is the distance between the restored image *u* and the observed image *f* where *h* is the PSF function and ${{{{\mathcal {T}}}}}$ is the degradation operator that encompasses Poisson and Gaussian noise. In what follows, we give the explicit expressions of the two terms.

**Data fidelity term**. A fidelity data term is generally derived from the general formation model, for instance dedicated to low photon counts and low-light regimes in fluorescence microscopy (e.g.,^15^). We formally demonstrate below that a conventional quadratic fidelity term, which is optimal when the images are corrupted by additive white Gaussian noise, is also appropriate for mixed Poisson-Gaussian noise.

Let us consider the following observation model^46^:

2 $$\begin{aligned} f(\textbf{x}) = v(\textbf{x}) + \eta \left(v(\textbf{x})\right) + \varepsilon (\textbf{x}), \end{aligned}$$

with $v(\textbf{x}) = (h *u)(\textbf{x})$, where $\textbf{x} \in \Omega$ is the pixel position in the domain $\Omega$, $\eta$ and $\varepsilon$ represent the signal-dependent Poisson noise component and the zero-mean Gaussian noise component respectively, such that:

3 $$\begin{aligned} \alpha \left(v(\textbf{x}) + \eta (v(\textbf{x}))\right) \sim {{\mathcal {P}}}(\alpha v(\textbf{x})), \quad \varepsilon (\textbf{x}) \sim {{\mathcal {N}}}(0, \tau ^2), \end{aligned}$$

$\alpha >0$ is the quantization factor of the photodetector, and $\tau ^2>0$ represents the Gaussian noise variance. According to the properties of Poisson distribution (${{\mathbb {E}}}[\cdot ]$ and $\text {Var}[\cdot ]$ denote the mathematical expectation and the variance respectively), we have

4 $$\begin{aligned} \left\{ \begin{aligned} {{\mathbb {E}}}\left[\alpha (v(\textbf{x}) + \eta (v(\textbf{x})))\right]&= \alpha v(\textbf{x}) = \text {Var}\left[\alpha (v(\textbf{x}) + \eta (v(\textbf{x})))\right] = \alpha ^2 \text {Var}\left[\eta (v(\textbf{x}))\right] \\ {{\mathbb {E}}}\left[\alpha (v(\textbf{x}) + \eta (v(\textbf{x})))\right]&= \alpha v(\textbf{x}) + \alpha {{\mathbb {E}}}\left[\eta (v(\textbf{x}))\right], \end{aligned} \right. \end{aligned}$$

which yields to ${{\mathbb {E}}}\left[\eta (v(\textbf{x}))\right] = 0$ and $\text {Var}\left[\eta (v(\textbf{x}))\right] = v{(\textbf{x}}) / \alpha$. Hence,

5 $$\begin{aligned} {{\mathbb {E}}}\left[f(\textbf{x})\right] = v(\textbf{x}) + \underbrace{{{\mathbb {E}}}\left[\eta (v(\textbf{x}))\right]}_{= 0} + \underbrace{{{\mathbb {E}}}\left[\varepsilon (\textbf{x})\right]}_{= 0}, \end{aligned}$$

and the overall variance of $f(\textbf{x})$ is the sum of $\text {Var}\left[\eta (v(\textbf{x}))\right]$ and $\text {Var}\left[\varepsilon (\textbf{x})\right] = \tau ^2$:

6 $$\begin{aligned} \text {Var}\left[f(\textbf{x})\right] = {{\mathbb {E}}}\left[(f(\textbf{x}) - {{\mathbb {E}}}[f(\textbf{x})])^2\right] = {{{\mathbb {E}}}\left[(f(\textbf{x}) - v(\textbf{x}))^2\right] = v{(\textbf{x}}) / \alpha + \tau ^2}. \end{aligned}$$

We conclude that $v(\textbf{x})$ is defined as the expected value of the noisy observations $f(\textbf{x})$ and $\text {Var}\left[f(\textbf{x})\right] = \alpha ^{-1} v{(\textbf{x}}) + \tau ^2$ is the noise variance^46–48^.

Assume an image $v = h *u$ corrupted by a mixed Poisson-Gaussian and denote *f* the noisy image. As explained above, the local noise variance can be represented at a given location $\textbf{x} \in \Omega$ as:

7 $$\begin{aligned} V_{noise}(\textbf{x}) \triangleq {{\mathbb {E}}}\left[(f(\textbf{x}) - (h *u)(\textbf{x}) )^2\right] = \alpha ^{-1} v(\textbf{x}) + \tau ^2. \end{aligned}$$

Let us assume that the average intensity is preserved, that is

8 $$\begin{aligned} \int _\Omega v(\textbf{x}) \ d\textbf{x} = \int _\Omega f(\textbf{x}) \ d\textbf{x}. \end{aligned}$$

It follows that, by pre-multiplying the intensities on both sides of the previous equation by a factor $\alpha ^{-1}$ and adding the constant value $\tau ^2$, we have:

9 $$\begin{aligned} \int _\Omega \alpha ^{-1} v(\textbf{x}) + \tau ^2 \ d\textbf{x} = \int _\Omega \alpha ^{-1} f(\textbf{x}) + \tau ^2 \ d\textbf{x}. \end{aligned}$$

Meanwhile, by integrating (7) over the image domain $\Omega$, we get

10 $$\begin{aligned} \int _\Omega {{\mathbb {E}}}\left[(f(\textbf{x}) - (h *u)(\textbf{x}))^2 \right] d\textbf{x}\;= \;& {} \int _\Omega \alpha ^{-1} v(\textbf{x}) + \tau ^2 d\textbf{x}, \nonumber \\ \int \int _\Omega (f\left(\textbf{x}) - (h *u')(\textbf{x})\right)^2 p(u'(\textbf{x})) \ d\textbf{x} \ du'(\textbf{x})\;=\; & {} \int _\Omega \alpha ^{-1} v(\textbf{x}) + \tau ^2 d\textbf{x}, \end{aligned}$$

where $p(u'(\textbf{x}))$ represents the probability distribution of *u*. Assume the following prior $p(u'(\textbf{x})) = {{{\textbf {1}}}}[f(\textbf{x}) = ((h *u)(\textbf{x}))]$ where ${{{\textbf {1}}}}[\cdot ]$ is the indicator function. Hence, we have by using (9)

11 $$\begin{aligned} \int _\Omega \left(f(\textbf{x}) - (h *u)(\textbf{x})\right)^2 d\textbf{x}= & {} \int _\Omega \alpha ^{-1} v(\textbf{x}) + \tau ^2 d\textbf{x}, \nonumber \\= & {} \int _\Omega \alpha ^{-1} f(\textbf{x}) + \tau ^2 d\textbf{x}, \nonumber \\ \Vert f - (h *u) \Vert _2^2\;= & \;\;\;{} \alpha ^{-1} \Vert f\Vert _1 + |\Omega | \tau ^2. \end{aligned}$$

Now, starting from the seminal paper^40^, the restored image is found by solving the following optimization problem (Poisson-Gaussian noise):

12 $$\begin{aligned} \min _u R(u) \quad \text {subject to} \quad \Vert f - (h *u) \Vert _2^2 \;=\; \alpha ^{-1} \Vert f\Vert _1 + |\Omega | \tau ^2. \end{aligned}$$

It turns out that (12) is a constrained formulation with an equality constraint which is not convex. The corresponding formulation with inequality constraint is

13 $$\begin{aligned} \min _u R(u) \quad \text {subject to} \quad \Vert f - (h *u) \Vert _2^2 \;\le \; \alpha ^{-1} \Vert f\Vert _1 + |\Omega | \tau ^2. \end{aligned}$$

It has been established that, under additional assumptions, that (12) and (13) are equivalent. A Lagrange formulation can be then derived since the the right-hand side of the inequality does not depend on the unknown image *u*:

14 $$\begin{aligned} \min _u R(u) + \lambda ' \Vert f - (h *u) \Vert _2^2, \end{aligned}$$

where the parameter $\lambda '>0$ balances the two energy terms and is an unknown function of the Poisson-Gaussian noise variance. The Karush-Tucker conditions guarantees that (15) and (13) are equivalent for a particular choice of $\lambda '$. Finally, if we set $\lambda '= \lambda ^{-1} \ne 0$, we can equivalently reformulate the minimization problem as:

15 $$\begin{aligned} \min _u \Vert f - (h *u) \Vert _2^2 + \lambda R(u). \end{aligned}$$

In conclusion, a quadratic fidelity term $F(\cdot )$ is appropriate for Gaussian-noise removal provided it is combined with regularizer $R(\cdot )$.

**Regularization term.** Commonly-used regularizers in image restoration have the following form:

16 $$\begin{aligned} R(u) = \int _{\Omega } \phi (Du(\textbf{x}))~d\textbf{x}, \end{aligned}$$

where *D* is a linear operator (called “regularization operator”) used to control the spatial distribution of *u* and $\phi (\cdot )$ is a positive potential function usually related to a norm distance. A typical example is the Tikhonov-Miller regularization^10^ defined as the squared norm of the gradient of *u*: $\phi (Du(\textbf{x})) = \Vert \nabla u(\textbf{x}) \Vert _2^2$.

**Table 2:** Properties of sparse regularizers.

Name	Regularizer $\phi (Du(\textbf{x}))$	Differentiation	Convexity	Smoothness order
$\text {TV-L}_{1,\rho }$^21^	$\rho \Vert \nabla u(\textbf{x}) \Vert _2 + (1-\rho ) |u(\textbf{x})|~d\textbf{x}$	1	Convex	Non smooth
GraphNet ($\text {GN}_\rho$)^17^	$\rho \Vert \nabla u(\textbf{x}) \Vert _2^2+ (1-\rho ) |u(\textbf{x})|$	1	Convex	Non smooth
Log Sparse Hessian Variation ($\text {LSHV}_\epsilon$)^16^	$\log (\epsilon + u^2(\textbf{x}) + \Vert {{{{\mathcal {H}}}}}u(\textbf{x})\Vert _F^2 )$	2	Non convex	Smooth
Sparse Variation ($\text {SV}_\rho$)^20^	$\sqrt{ \rho ^2 \Vert \nabla u(\textbf{x})\Vert _2^2 + (1 - \rho )^2 u(\textbf{x})^2\ }$	1	Convex	Non smooth

We propose a convex sparsity-promoting regularizer based on second-order derivatives to avoid the emergence of undesirable stair-casing effects. The Sparse Hessian Variation (SHV) regularizer is defined as follows

17 $$\begin{aligned} {\text {SHV}}_{\rho }(u) = \int _{\Omega } ~\underset{\Vert D_{_{2, \rho }} u(\textbf{x}) \Vert _2}{\underbrace{\sqrt{ \rho ^2 \Vert {{\mathcal {H}}} u(\textbf{x})\Vert _F^2 + (1 - \rho )^2 u(\textbf{x})^2~}}}~d\textbf{x}\, \end{aligned}$$

where $\Vert \cdot \Vert _F$ denotes the matrix Frobenius norm and $\rho \in [0, 1]$ is the weighting parameter. Moreover, since the Frobenius norm of the Hessian matrix is equal to the Euclidean norm of its vectorized version, the operator $D_{2,\rho } \in {{\mathbb {R}}}^{10}$ is defined (for $d=3$) as:

18 $$\begin{aligned} D_{_{2, \rho }}u(\textbf{x}) \triangleq \left( (1 - \rho ) u(\textbf{x}), \rho ~\frac{\partial ^2 u(\textbf{x})}{\partial _{xx}}, \rho ~\frac{\partial ^2 u(\textbf{x})}{\partial _{yy}}, \rho ~\frac{\partial ^2 u(\textbf{x})}{\partial _{zz}}, \rho ~\frac{\partial ^2 u(\textbf{x})}{\partial _{xy}}, \rho ~\frac{\partial ^2 u(\textbf{x})}{\partial _{xz}}, \rho ~\frac{\partial ^2 u(\textbf{x})}{\partial _{yz}}, \rho ~\frac{\partial ^2 u(\textbf{x})}{\partial _{yx}}, \rho ~\frac{\partial ^2 u(\textbf{x})}{\partial _{zx}}, \rho ~\frac{\partial ^2 u(\textbf{x})}{\partial _{zy}} \right) ^T. \end{aligned}$$

The idea behind this combination is to sparsify jointly the spatial distribution of image intensities and the second order derivatives of the image to encourage smooth variations between spatially-contiguous non-zero regions of the underlying image. The resulting image contain bright objects against a large smooth dark background, and no spurious edge. Unlike LSHV^16^, SHV is convex and more flexible as it involves a parameter $\rho \in [0,1]$ which is helpful to eliminate or preserve background. In SPITFIR(e), we consider only three values to control the amount of sparsity: $\rho = 0.1$ (“sparse”), $\rho = 0.6$ (“moderately” sparse), and $\rho = 0.9$ (“weakly” sparse). It is worth noting that, unlike conventional energy modelings in fluorescence microscopy, the background is not preliminarily computed with an ad-hoc method beforehand but is implicitly obtained when *u* is estimated.

**Discrete formulation and optimization.** We present here the discretization setting required to derive the optimization algorithm. Let us consider a sampling grid of the following form in 3D ($d=3$) (expressions in 2D are similar).

$$\begin{aligned} \Lambda = {{\mathbb {Z}}}^3 \cap \Omega = \lbrace 1, 2, \ldots , N_x \rbrace \times \lbrace 1, 2, \ldots , N_y \rbrace \times \lbrace 1, 2, \ldots , N_z \rbrace . \end{aligned}$$

The observed noisy and blurry image *f* is represented by its digitized (discrete) version as follows: $f ={{{{\mathcal {T}}}}}(Hu)$, where $f, u \in {{\mathbb {R}}}_+^{N}$ with $N = N_x \times N_y \times N_z$, $H \in {{\mathbb {R}}}^{N \times N}$ is a matrix that models the point spread function of the microscope in the discrete setting, and ${{{{\mathcal {T}}}}}$ is degradation operator. Both *u* and *f* are assumed to be non negative. In the discrete setting, the blurring operator *H* corresponds to a discrete convolution which can be efficiently computed by using fast Fourier transform (FFT)^49–52^. To discretize $D_{_{2, \rho }}$, we use standard finite differences for the Hessian operators with Neumann conditions on image boundaries. The 2D-3D deconvolution problem is then defined in the discrete setting as the minimizer of the following energy:

19 $$\begin{aligned} E(u) = \dfrac{1}{2} \Vert Hu - f\Vert _2^2 + \lambda \Vert D_{2, \rho }u \Vert _2 + \imath _{{\varvec{{{\mathcal {C}}}}}}(u), \end{aligned}$$

where $\lambda >0$ is the regularization parameter and $\imath _{{\varvec{{{\mathcal {C}}}}}}$ is the characteristic function of the convex subset ${\varvec{{{\mathcal {C}}}}}$ defined as: $\imath _{{\varvec{{{\mathcal {C}}}}}} = 0$ if $u \in {\varvec{{{\mathcal {C}}}}}$ and $+ \infty$ otherwise, is very helpful to impose positivity constraint on the solution.

The optimization problem (19) is convex since the underlying energy functional is defined as the sum of convex terms, but it is non-smooth. To minimize the energy (19), several algorithms have been investigated, including FISTA^53^, ADMM^24,25^ and MM^54^ algorithms. Instead of applying the aforementioned optimization methods, we investigated a first-order method to minimize the sum of convex functions, based on the proximal splitting approaches^28,29,55–60^. The main idea consists in splitting the original problem into several simple sub-problems in the way that each single function of the sum can be processed separately (see details in Appendix). These operators are well-suited for large-scale problems arising in signal and image processing, because they only exploit first-order information of the function and thus enable fast and efficient computation. To solve the minimization problem (19), we adapted the full splitting approach described in^28,29^. The key idea is to evaluate the gradient, proximity and linear operators individually in order to avoid implicit operations such as inner loops or inverse of linear operators.

**Selection of the regularization parameter**. The adjustment of the regularization parameter $\lambda$ in (19) is generally crucial in most image restoration algorithms, and may be time consuming. A non optimal choice of this parameter may over-smooth object borders, suppress structural details, generate artifacts or weakly reduce noise. The practical issue is then to automatically tune this parameter on a case-by-case basis to get the best performance as possible, from the input noisy image.

Instead of applying the usual principle of cross-validation, we investigated another strategy which is based on the comparison of several competing restored images (or regions of interest (ROIs)) over a range of parameters $\lambda$. All the intensity values are preliminary normalized with respect to the maximum intensity to get intensity values in the range [0, 1]. Henceforth, the energy (1) is rewritten as follows:

20 $$\begin{aligned} E_{\lambda ^\prime }(f, u) = \lambda ^\prime F(f,u; h, {{\mathcal {T}}}) + (1- \lambda ^\prime ) R_\rho (u) \end{aligned}$$

where $\lambda ^\prime \in ]0, 1[$ is a positive constant such that $\lambda ^\prime = (1+\lambda )^{-1}$. The two energy terms are expected be equally balanced if $\lambda ^\prime = 1/2$, which is considered as the default value in the algorithm. Nevertheless, we applied the minimax principle^61^ to find $u^\star$ that best minimizes, over all $\lambda ^\prime$ values, the maximum of $E_{\lambda ^\prime }(f, u)$:

$$\begin{aligned} (u^\star , {\lambda ^\prime }^\star ) = \min _{u} ~\max _{\lambda ^\prime } \; E_{\lambda ^\prime }(f, u) = \max _{\lambda ^\prime } \min _{u} E_{\lambda ^\prime }(f, u) ~{\mathop {=}\limits ^{\triangle }}~ \max _{\lambda ^\prime } E_{\lambda ^\prime }(f, u^\star ). \end{aligned}$$

The optimization problem may be solved by examining the finite set of solutions $E_{\lambda ^\prime }(f, u^\star )$ and selecting the $\lambda ^\prime$ valued associated to the highest energy $E_{\lambda ^\prime }(f, u^\star )$. he good news is that Gennert and Yuille^61^ demonstrated that there exists a unique value $\lambda ^\prime$ that maximizes $E_{\lambda ^\prime }(f, u^\star )$ (convexity of $E_{\lambda ^\prime }(f, u^\star )$). Hence, by initializing $\lambda ^\prime = 1/2$ and considering a finite set $\{\lambda ^\prime _m\}_{m=1}^M$ of increasing values such that $\lambda ^\prime _{\lfloor M/2 \rfloor } = 1/2$, a fast search technique can be developed which consists in restoring the image with $\lambda ^\prime = \lambda ^\prime _{\lfloor M/2 \rfloor +1}$ and $\lambda ^\prime _{\lfloor M/2 \rfloor -1}$ and keeping the $\lambda ^\prime$ corresponding to the highest energy $E_{\lambda ^\prime }(f, u^\star )$. The raw image is then restored with a highest (or lowest value) of $\lambda ^\prime$, and the SPITFIR(e) procedure is repeatedly applied on raw data (with $\lambda = (1-\lambda ^\prime )/\lambda ^\prime$) until the energy $E_{\lambda ^\prime }(f, u^\star )$ decreases (or increases).

The optimal value solution $(u^\star , {\lambda ^\prime }^\star )$ is very helpful as it corresponds to the smoothest image that is acceptable (before over-smoothing) to the user. As illustrated in Fig. 6(**a**), we found that the optimal parameter $\lambda ^{\prime ^*}$ varies little in ROIs ($\lambda {^\prime }^*\in [0.2, 0.4]$ in Fig. 6(a)), including if the ROI is the entire image. This means that the ROI can be the entire image or a small patch containing meaningful fluorescent signals and manually selected by the practitioner. The aforementioned procedure is then run on a ROI (2D or 3D patch) by considering three levels of sparsity (“high”, “moderate”, “weak”) given a finite set of values of $\lambda ^\prime$. This preview study is very low time consuming and computationally demanding. The results are displayed as a $3 \times 3$ matrix whose the rows and columns represent smoothness and sparsity (see Fig. 6(b)).

The practitioner then selects a given element in this $3 \times 3$ matrix to modify or to confirm the default parameter settings. In general, the 3x3 matrix summarizes the possible solutions, quantified on 3 levels for each parameter. This visualization is optional but is helpful to typically confirm the amount of sparsity and smoothness in the reconstructed images before processing large volumes.In a more automatic mode, the expert only specifies if the expected image is “highly”, “moderately” or “weakly” sparse, without using the visualization module. The smoothing parameter is then automatically estimated following the minimax principle. To our knowledge, there is few practical rules to automatically set the two parameters to process 3D images and videos; an alternative approach to estimate algorithm parameters consists in computing FRC curves from a single image and subsampling operations, after each iteration of the algorithm (e.g, RL algorithm)^27^.

### Implementation details and code availability

The input image is the raw (noisy and blurred) image. In the case of Lattice Light Sheet Microscopy, a necessary “deskew” operation is applied to raw data before to be supplied to SPITFIR(e).

Images have been processed on classical workstation for CPU calculation (CPU Intel Xeon 2.8GHz 4 threads). We use this setup since it is the most common setup in biology labs. For high speed processing we developed a GPU version of SPITFIR(e). We run the GPU on two types of GPU, a Nvidia Quadro M2000 that can be found in most workstations and a Nvidia Tesla K80 for computing grid. Typical computing times (CPU and GPU) for processing 2D-3D-4D images are reported in the Table 3. These computing times are small and take into account the full data processing steps (including data normalization and data copy to the GPU device). The code includes several CPU and GPU implementations and can be downloaded for free from the GitHub website (https://github.com/sylvainprigent/spitfire) along with accompanying documentation .

**Table 3:** Computing times of SPITFIR(e) (200 iterations for energy minimization).

SPITFIR(e)	Image size	CPU	GPU	
200 iterations		Intel Xeon (2.8 GHz, 4 threads)	Nvidia Quadro M2000	Nvidia Tesla K80
2D Denoising	$512\times 512$	349 ms	345 ms	278 ms
3D Denoising	$512\times 512\times 22$	8 s 74 ms	3 s 78 ms	1s 277ms
4D Denoising	$512\times 512\times 14\times 32$	8 min 23 s 789 ms	1 min 40 s 98 ms	45 s 744 ms
2D Deconvolution	$512\times 512$	767 ms	550 ms	428 ms
3D Deconvolution	$512\times 512\times 22$	23 s 574 ms	5 s 786 ms	2 s 713 ms

### Cell culture fluorescence labeling

The hTERT-immortalized RPE1 cells (Human Retinal Pigment Epithelial Cell) were purchased from ATCC (CRL-4000). RPE1 cells within 30 passage number were used and grown in DMEM-F12 medium without phenol red (Life Technologies) supplemented with 10% (vol/vol) fetal bovine serum (FBS) at 37$^{\circ }$C in humidified atmosphere with 5% CO2. For LLSM (Lattice Light Sheet Microscopy) imaging, cells were seeded 5 to 6 hours before image acquisition at $150 \times 10^3$ per well of a 6 well plate, containing 3 to 4 coverslips with a 5mm diameter, treated as previously described in Chen *et al.*^30^. Live cells were stained for microtubule and mitochondria using the Tubulin Tracker DR (Tub-Tracker Deep Red, ThermoFisher Scientific) probe (Ex. 652nm/Em. 669) at 10 ng/ml and 100ng/ml of PKMR (PK Mito Red), a cyclooctatetraene-conjugated-cyanine-3 dye (Ex. 549/Em. 569) kindly provided by Dr. Z. Chen from Peking University^31^, respectively. Cell labeling was performed in two steps. Cells were first incubated for 15min with Tubulin Tracker DR and washed twice with LLSM medium (DMEM/F-12, without phenol red, with 1% BSA, 1mM pyruvate, and 20mM HEPES). Coverslips were then transferred to the lattice light-sheet microscope (LLSM) sample holder and inserted into the imaging chamber containing 3.5 ml of the later medium and 0.1 mg/ml of PKMR.

Previously described Hela cells expressing Rab5-eGFP, CRISP genome edited SUM 159 breast carcinoma cell line, using eGFP-tagged $\sigma$ unit of the AP2 complex (Adaptor Protein complex) were kindly provided by Dr. Tomas Kirchhausen (HMS, Boston, MS, USA) via Dr Ludger Johannes (Institut Curie, Paris, France), and CD-M6PR-EGFP expressing Hela cell line, by Dr Bernard Hoflack (TU-Dresden, Dresden, Germany). Human retinal pigment epithelial cells (RPE1) expressing mCherry-LifeAct were kindly provided by Dr Manuel Thery^62^. All these cells were cultured and prepared for imaging with the LLSM as described above.

For fixed samples, RPE1 cells were cultured and seeded as above, incubated for two additional hours with $1\mu \hbox {l}/\hbox {ml}$ of SiR-Actin (from a stock solution at 1mM; Ex.652/Em.674; Spirochorme AG, SWZ) and $1\mu \hbox {l}/\hbox {ml}$ of verapamil (from a stock solution at 1 mM) before washing in PBS and fixation with 4% paraformaldehyde freshly prepared in PBS from a 16% stock solution (Electron Microscopy Science, PA, USA). Cells were then permeabilized with 0.2% TX100 for 10 minutes, before incubation with anti $\alpha$-tubulin DM1 IgG1-mAb for 45 minutes, diluted at 1/500 in PBS plus 0.1% FBS. After washing, goat anti-mouse IgGs secondary antibodies coupled to Alexa 488nm (diluted at 1/200 in PBS plus 0.1% FBS; Ex.496/Em.519; ThermoFisher scientific, Bordeaux, France) were added for 30 minutes incubation. Coverslips were preserved at $4^\circ \hbox {C}$ in PBS before transfer to the LLSM chamber containing 3.5 ml of the PBS at the room temperature.

For STED imaging RPE1 cells were plated on glass bottom Petri dishes ($\mu$-dish 35mm-High,1.5H, Ibidi, GmbH, Grafelfing, Germany). Microtubule labeling was performed as for LLSM imaging, while mitochondria were labeled by diluting 16000 $\times$ a 1mM stock solution of PKMITO Orange [gifted by Dr. Z. Chen from Peking University and Genvivo Biotech, Beijing, PR of China] in DMEM/F12 medium for a final 62.5 nM, for 15 minutes at $37^\circ \hbox {C}$ before image acquisition.

For MFM imaging the U2OS cells, originating from human bone osteosarcoma with an epithelial morphology (obtained from ATCC (HTB-96)), were grown in DMEM culture medium (11880, by Thermo Fisher Scientific) without phenol red supplemented with 1% Glutamax, 1% Penicillin-Streptomycin and 10% fetal bovine serum (26140079, by Thermo Fisher Scientific). Cells are kept at $37^\circ$C in the presence of 5% CO2 in a humid environment. Cells were seeded on 25mm round coverglass for 12h in the same culture conditions. Cells were then transfected with TOM20-GFP DNA plasmid using X-tremeGENE HP DNA Transfection Reagent (Sigma). After 24 hours, cells are mounted and imaged. During the experiments cells were kept at $37^\circ$C with 5% CO2 in a humid environment using a Tokai Hit heating system (INUBG2E-PPZI).

Previously acquired datasets of M10 cells, stably expressing Langerin tagged with eYFP, or Rab11a tagged with mCherry^38^ or transiently transfected with PhLuorin Transferrin-Receptor (TfR)^37^ were also exploited in this study.

### Microscopy

**Lattice light-sheet microscopy**. LSSM was done on a commercialized version of a previously described setup^30^ from 3i (Denver, USA). Cells were scanned incrementally through a 20 $\mu$m long light sheet in 600 nm steps using a fast piezoelectric flexure stage equivalent to $\simeq$ 325 nm with respect to the detection objective and were imaged using a sCMOS camera (Orca-Flash 4.0; Hamamatsu, Bridgewater, NJ). Excitation was achieved with 488 nm (Sapphire Coherent), 560 nm (MPB Communications) or 642 nm (MPB Communications) diode lasers at 10-20% acousto-optic tunable filter transmittance with 50-200 mW (initial box power) through an excitation objective (Special Optics 28.6$\times$ 0.7 NA 3.74-mm water-dipping lens) and detected via a Nikon CFI Apo LWD 25$\times$ 1.1 NA water-dipping objective with a 2.5$\times$ tube lens with a final pixel size of 104 nm. Lattice light-sheet imaging was performed using an excitation pattern of outer NA equal to 0.55 and inner NA equal to 0.493. Composite volumetric datasets were obtained using $\simeq$10 ms exposure/slice/channel at a time resolution of 2 seconds per total cell volume (about 60 planes). Fifty-six time points were acquired within 3 to 4 minutes (total raw data, only 1:45 min were analyzed for the movie) for live cell acquisition. Ten time-points were acquired within 15 to 20 seconds for fixed cell acquisition. Acquired data were deskewed, a necessary step to realigned image frames, using LLSpy, a python library to facilitate lattice light sheet data processing (copyright to T. Lambert, Harvard Medical School, Boston, USA; https://github.com/tlambert03/LLSpy). Deskewed images are then considered as Raw images. Napari, a multi-dimensional image viewer for Python (https://github.com/napari/napari), was used for 3D rendering. Maximum intensity projections were generated using ImageJ/Fiji 1.53c^63^. Intensity profiles were plotted using Matlab2019b. Related supplementary Videos S1 and S2 were generated using napari and napari-animation plugin (https://github.com/napari/napari-animation). 3D Richardson-Lucy deconvolution was performed using deconvlucy from MATLAB imaging processing toolbox. Fourier Image Resolution (FIRE) was estimated using Fourier Ring Correlation^32^.

**Stimulated emmission depletion microscopy (STED).** Image acquisition was performed with a STEDYCON module (Abberior Instruments, Göttingen, Germany) mounted at the camera port TCS SP8 STED microscope (Leica, Mannheim, Germany) with a HC PL APO C2S $\times 100$ oil objective (1.4 NA) used in 2D mode. Depletion was performed with a 775 nm pulsed laser 1-7 ns at 80% (413 mW at the objective lens position). Labeled mitochondria and microtubules were imaged with excitation at a wavelength of 594 nm and 640 nm, respectively. Nominal laser powers were adjusted at 7% for the 594 nm laser ($3.15 \,\mu W$ at the objective lens position) and at 10% for the 640 nm ($26 \,mW$ at the objective lens position). The time-gated fluorescence detection was done on a detector single photon counting avalanche photodiode between 578–627 nm and 650–700 nm with a pinhole settled at 1.1 AU. Imaging was executed with the 9 lines accumulation. Pixel dwell time was $10 \,\mu s$ and a pixel size of $25 \,\hbox {nm} \times 25 \,\hbox {nm}$. Images were generated using ImageJ/Fiji 1.53c3. Line profiles plots were measured with ImageJ. Then line profiles were fitted to a Gaussian model using Matlab2019b. Finally, Full width at half maximum (FWHM) is estimated from the fitting results. Unpaired two-sample *t*-test and variance *F*-test were performed using GraphPad Prism 9. 2D Richardson-Lucy deconvolution was performed using deconvlucy from MATLAB imaging processing toolbox.

**Multifocus microscopy (MFM).** Imaging was performed on a custom made MFM system with a Nikon 100x oil immersion (1.4 NA) detection objective. We recall briefly the main characteristics of the system^64^. The emission path of a wide-field microscope (Nikon Ti) was modified by introducing several optical elements. At the output of the microscope a lens conjugated the back focal plane of the microscope to a multifocus grating (MFG). The motif of the phase grating is designed such that it splits with high efficiency the emission into 9 diffraction orders with equal intensities. The periodicity of the grating is distorted in order to add a different defocusing powers in the different diffraction orders. A chromatic correction module (CCM) based on the combination of a multifaceted prism and gratings corrects the chromatic aberration introduced by the MFG. A final lens is placed before the imaging camera (iXon Ultra897, Andor) in order to form the final images. Each diffraction order is focused on a different part of the camera, and yields an image of a different axial plane of the sample. As such, a single camera exposure results in a simultaneous acquisition of 9 focal planes. For live cell imaging, a 488nm laser was used to excite the sample. The fluorescence was filtered by a fluorescence filter (FF01-525/45-25, Semrock). The exposure time of the camera was set to 50ms and the laser power was adjusted to yield different signal to noise ratio.

**Other imaging modalities.** Fast confocal imaging in Fig. 4 and Supplementary Figure. S4,S5 was performed with a spinning-disk confocal based on a Ti-Eclipse inverted microscope (Nikon, Tokyo, JP) equipped with X1 confocal head (Yokogawa, Tokyo, JP), Prime95B, an sCMOS camera (Teledyne Photometrics, Tucson, AZ). Thirty z-stack cell volumes were obtained, scanning incrementally in 300 nm z-step at 20 ms/frame (14 frames/volume), using a 100X, Plan Apo NA 1.4 oil objective (Nikon, Tokyo, JP) with a final pixel size of 110 nm. Excitation was achieved at 488-nm (100 mW) with a diode laser (GATACA systems, Massy, FR), at different illuminations, adjusting the acousto-optic tunable filter transmittance at 80% (I), 40%(I/2) and 20%(I/4). The acquisition is driven by the Metamorph 8.6 software (Molecular Devices, San Jose, CA). The original single 2D image in Fig. 5 was extracted from a previously acquired 3D+Time stack (512 $\times$ 256 $\times$ 1 plane $\times$ 74 frames) by laser line scanning microscopy (A1R, Nikon, Tokyo, JP) using a $\times$100, NA 1.35, APO lambda S silicone objective and excitation with a 561 nm laser (by courtesy of the PICT-Curie Imaging platform) and further denoised using nDSafir^2^ [5 iterations and patch size of 11 $\times$ 11 pixels], in order to build a reference “ground truth”. Spinning-disk confocal microscopy (CM) and Total Internal Reflection microscopy (TIRFM) of Table 1 and Fig. 5 were either used as described before in Gidon *et al.*^38^, Pécot *et al.*^65^ or previously acquired datasets^37,66^, reused in this study.

## Supplementary Information

APPENDIX (Technical details of the discrete formulation and optimization) and SUPPLEMENTARY FIGURES.

SUPPLEMENTARY VIDEO S1. Composite movie corresponding to the time series of insets in Fig. 2 a,b. Upper part from left to right, microtubules, mitochondria and superimposed MIP data from LLSM after deskew treatment. Lower part, similar disposition after SPITFIR(e) 4D denoising and 3D deconvolution. Only 1:45 min of a 4 min series is shown. Scale bar: $5 \,\mu \hbox {m}$.

SUPPLEMENTARY VIDEO S2. Multi-Angle view of the 3D of superimposed image stacks from the same time series as in Supplementary Video 1. Upper part data from LLSM after deskew treatment, lower part after SPITFIR(e) 4D denoising and 3D deconvolution. Scale Bar = $5 \,\mu \hbox {m}$.

## Supplementary Information

Supplementary Information 1.

Supplementary Information 2.

Supplementary Information 3.

## Data Availability

Data generated during this study are available in figShare: 10.6084/m9.figshare.21378327.
