# Supplementary material for: SPITFIR(e): a supermaneuverable algorithm for fast denoising and deconvolution of 3D fluorescence microscopy images and videos
Source: Sci Rep. 2023 Jan 27;13:1489. doi: 10.1038/s41598-022-26178-y (PMC9883505; doi:10.1038/s41598-022-26178-y)
Supplement: Supplementary file 1 — Supplementary Information 1. [file 41598_2022_26178_MOESM1_ESM.pdf]

SUPPLEMENTARY INFORMATION

SPITFIR(e): A supermaneuverable algorithm for fast denoising and deconvolution of 3D fluorescence microscopy images and videos

Sylvain Prigent[1,2,+], **Hoai-Nam Nguyen** [1,2,+], **Ludovic Leconte**[1,2], **Cesar Augusto Valades-Cruz**[1,2], **Bassam Hajj**[3], **Jean Salamero**[1,2], **Charles Kervrann**[1,2,*]

[1] SERPICO Project-Team, Inria Centre Rennes-Bretagne Atlantique, 35042, Rennes Cedex, France
[2] SERPICO/STED Team, UMR144 CNRS Institut Curie, PSL Research University, Sorbonne Universités, 75005, Paris, France
[3] Laboratoire Physico-Chimie, Institut Curie, PSL Research University, Sorbonne Universités, CNRS UMR168, 75005, Paris, France
[*] charles.kervrann@inria.fr
[+] these authors contributed equally to this work.

APPENDIX

Discrete formulation

The observed noisy and blurry image f is represented by its digitized (discrete) version and not by its continuously defined counterpart. The continuous model is not appropriate for discrete images even though the estimation of the continuous image u from discrete samples of f is in principle possible. Consider a discrete formulation by assuming that the images u and f are non negative and sampled according to the sampling grid

$$\Lambda = \mathbb{Z}^2 \cap \Omega = \{1,2,\dots,N_x\} \times \{1,2,\dots,N_y\} \times \{1,2,\dots,N_z\}. \tag{21}$$

The observed noisy and blurry image f is represented by its digitized (discrete) version as follows:

$$f = \mathscr{T}(h * u)$$

where $f, u \in \mathbb{R}_+^N$ with $N = N_x \times N_y \times N_z$, $H \in \mathbb{R}^{N\times N}$ is a matrix that models the point spread function of the microscope in the discrete setting, and $\mathscr{T}$ is the degradation operator. For a coordinate $(i,j,k) \in \Lambda$, we denote by $u_{i,j,k}$ (resp. $f_{i,j,k}$) the value of u (resp. f) at position $(i,j,k) \in \Lambda$. A discrete version of these images are therefore given by $\{u_{i,j,k}\}_{1\le i\le N_x, 1\le j\le N_y, 1\le k\le N_z}$ and $\{f_{i,j,k}\}_{1\le i\le N_x, 1\le j\le N_y, 1\le k\le N_z}$. Denote $\mathscr{X} = \mathbb{R}^N$ with $N = N_x \times N_y \times N_z$, a finite dimensional vector space equipped with a standard inner (scalar) product

$$\langle w, w' \rangle_{\mathscr{X}} = \sum_{i=1}^{N_x} \sum_{j=1}^{N_y} \sum_{k=1}^{N_z} w_{i,j,k} w'_{i,j,k}. \tag{22}$$

The induced norm by the defined inner product is given by

$$\|w\|_{\mathscr{X}_2} = \sqrt{\langle w, w \rangle_{\mathscr{X}}} = \left(\sum_{i=1}^{N_x} \sum_{j=1}^{N_y} \sum_{k=1}^{N_z} w^2_{i,j,k} \right)^{\frac{1}{2}}. \tag{23}$$

In the discrete setting, the blurring operator H corresponds to a discrete convolution which can be efficiently computed by using fast Fourier transform (FFT)[49–52]. To discretize $D_{2,\rho}$, we use standard finite differences to approximate the second derivatives along the three dimensions of the volume at voxel (i,j,k), with Neumann conditions on image boundaries:

$$(D_{2,\rho} u)_{i,j,k} = \begin{pmatrix} (1-\rho) u_{i,j,k} \\ \rho (\Delta^{(2)}_{1,1} u)_{i,j,k} \\ \rho (\Delta^{(2)}_{2,2} u)_{i,j,k} \\ \rho (\Delta^{(2)}_{3,3} u)_{i,j,k} \\ \rho (\Delta^{(2)}_{1,2} u)_{i,j,k} \\ \rho (\Delta^{(2)}_{1,3} u)_{i,j,k} \\ \rho (\Delta^{(2)}_{2,3} u)_{i,j,k} \\ \rho (\Delta^{(2)}_{2,1} u)_{i,j,k} \\ \rho (\Delta^{(2)}_{3,1} u)_{i,j,k} \\ \rho (\Delta^{(2)}_{3,2} u)_{i,j,k} \end{pmatrix} \in \mathbb{R}^{10}, \tag{24}$$

where

$$(\Delta^{(2)}_{1,1} u)_{i,j,k} = \begin{cases} u_{i+1,j,k} - 2u_{i,j,k} + u_{i-1,j,k} & \text{if } 1 < i < N_x, \\ 0 & \text{otherwise,} \end{cases}$$

$$(\Delta^{(2)}_{2,2} u)_{i,j,k} = \begin{cases} u_{i,j+1,k} - 2u_{i,j,k} + u_{i,j-1,k} & \text{if } 1 < j < N_y, \\ 0 & \text{otherwise,} \end{cases}$$

$$(\Delta^{(2)}_{3,3} u)_{i,j,k} = \begin{cases} \delta^2 (u_{i,j,k+1} - 2u_{i,j,k} + u_{i,j,k-1}) & \text{if } 1 < k < N_z, \\ 0 & \text{otherwise,} \end{cases}$$

$$(\Delta^{(2)}_{1,2} u)_{i,j,k} = (\Delta^{(2)}_{2,1} u)_{i,j,k} = \begin{cases} (u_{i+1,j+1,k} - u_{i+1,j,k} - u_{i,j+1,k} + u_{i,j,k}) \\ \qquad \text{if } i < N_x, j < N_y \text{ and } k < N_z, \\ 0 \qquad \text{otherwise.} \end{cases}$$

$$(\Delta^{(2)}_{1,3} u)_{i,j,k} = (\Delta^{(2)}_{3,1} u)_{i,j,k} = \begin{cases} \delta (u_{i+1,j,k+1} - u_{i+1,j,k} - u_{i,j,k+1} + u_{i,j,k}) \\ \qquad \text{if } i < N_x, j < N_y \text{ and } k < N_z, \\ 0 \qquad \text{otherwise.} \end{cases}$$

$$(\Delta^{(2)}_{2,3} u)_{i,j,k} = (\Delta^{(2)}_{3,2} u)_{i,j,k} = \begin{cases} \delta (u_{i,j+1,k+1} - u_{i,j+1,k} - u_{i,j,k+1} + u_{i,j,k}) \\ \qquad \text{if } i < N_x, j < N_y \text{ and } k < N_z, \\ 0 \qquad \text{otherwise.} \end{cases}$$

and δ is the ratio of the lateral-to-axial step sizes.

The discrete operators can be used to define the discrete SHV regularizer as:

$$\|D_{2,\rho}u\|_2 = \sum_{i=1}^{N_x}\sum_{j=1}^{N_y}\sum_{k=1}^{N_z} \|(D_{2,\rho}u)_{i,j}\|_2, \tag{26}$$

where the L_1-norm acts now on the discrete domain Λ. The 3D deconvolution problem is defined in the discrete setting as the minimizer of the following energy:

$$E(u) = \frac{1}{2}\|Hu-f\|_2^2 + \lambda\|D_{2,\rho}u\|_2 + \iota_{\mathscr{C}}, \tag{27}$$

where $\lambda > 0$ is the regularization parameter and $\iota_{\mathscr{C}}$ is the indicator of a convex set $\mathscr{C}$ such as:

$$\mathscr{C} = \{u : \underline{u}_{i,j,k} \leq u_{i,j,k} \leq u_{\max},\ 1 \leq i \leq N_x,\ 1 \leq j \leq N_y,\ 1 \leq k \leq N_z\}, \tag{28}$$

where the upper bound $u_{\max} > 0$ is the maximal intensity value allowed and $\underline{u}_{i,j,k} \geq 0$ is an estimated lower bound of the pixel intensity which is spatially varying and then adapted to each pixel location. The spatially varying constraint on the lower bound of pixel intensity not only guarantees positivity of the solution but also helps to avoid over-sparsifying effect. In our experiment, for the sake of simplicity, we use the following lower bound $\underline{u}_{i,j,k} = \max(0, \tilde{f}_{i,j,k} - c\tau)$ where $c > 0$ and $\tilde{f}$ is a smoothed version of the observed noisy image f by a low-pass (Gaussian) filter.

Energy minimization and splitting algorithms

We notice that the objective function

$$E(u) = \frac{1}{2}\|Hu-f\|_2^2 + \lambda\|D_{2,\rho}u)\|_2 + \iota_{\mathscr{C}}, \tag{29}$$

is a sum of linear composite functions as $u \mapsto \sum_{n=1}^{3}\mathscr{F}_m(L_m u)$, where each $\mathscr{F}_m$ is a convex function and each L_m is a linear bounded operator. Formally, we can write $\mathscr{F}_1 = \iota_{\mathscr{C}}$, $L_1 = \mathrm{Id}$, $\mathscr{F}_2 = \lambda\|\cdot\|_2$, $L_2 = D_{2,\rho}$ and $\mathscr{F}_3 = \frac{1}{2}\|\cdot\|^2$, $L_3 = H(\cdot) - f$. Generic primal-dual proximal approaches can be used to minimize this linear combination of convex functions as proposed in[67,68], but it is not optimal since the smoothness of the quadratic terms $\frac{1}{2}\|Hu-f\|_2$ is not exploited. In order to solve the problem (29), the design of an appropriate algorithm requires therefore to take into account the specific form of the corresponding energy, i.e., the sum of a simple convex function $\mathscr{F} = \iota_{\mathscr{C}}$, a more sophisticated composite function $\mathscr{G} \circ L = \lambda\|D_{2,\rho}(\cdot)\|_2$ (here, $\mathscr{G} = \lambda\|\cdot\|_2$ and $L = D_{2,\rho}$) and a smooth function $\mathscr{H} = \frac{1}{2}\|H(\cdot) - f\|_2$.

In what follows, we present a first-order method to minimize the sum of convex functions based on the proximal splitting approaches[28,29,55–60]. It consists in decomposing (splitting) the original problem into several simple sub-problems in the way that each single function of the sum can be processed separately. Indeed, smooth function involves its gradient operator, while non-smooth function implies its Moreau proximity operator[69]. These operators are well-suited for large-scale problems arising in signal and image processing, because they only exploit first-order information of the function and thus enable fast and efficient computation.

Let us recall first that the proximity operator of a convex function $\mathscr{J} : \mathbb{R}^N \to \mathbb{R}$ is defined as

$$\mathrm{prox}_{\tau\mathscr{J}}(u) = \arg\min_{w\in\mathbb{R}^N}\ \mathscr{J}(w) + \frac{1}{2\tau}\|u-w\|_2^2\ , \tag{30}$$

where $\tau > 0$ is a control parameter. From this definition, it easy to verify that the proximity operator of the function $\mathscr{F}(u) = \iota_{\mathscr{C}}(u)$ is nothing else than the projection onto the convex subset $\mathscr{C}$ as the following

$$\begin{aligned}\mathrm{prox}_{\tau\mathscr{F}}(u) &= \arg\min_{w\in\mathbb{R}^N}\ \iota_{\mathscr{C}}(w) + \frac{1}{2\tau}\|u-w\|_2^2 \\ &= \arg\min_{w\in\mathscr{C}}\ \|u-w\|_2^2.\end{aligned} \tag{31}$$

If we denote $\mathrm{proj}_{\mathscr{C}}$ the projection operator on $\mathscr{C}$, its closed-form expression is given by

$$\left(\mathrm{proj}_{\mathscr{C}}(u)\right)_{i,j,k} = \max(\underline{u}_{i,j,k}, \min(u_{\max}, u_{i,j,k})). \tag{32}$$

Moreover, the quadratic function $\mathscr{H}(u) = \frac{1}{2}\|Hu-f\|_2^2$ possesses an analytic form for its associated proximity operator

$$\begin{aligned}\mathrm{prox}_{\tau\mathscr{H}}(u) &= \arg\min_{w\in\mathbb{R}^N}\ \frac{1}{2}\|Hw-f\|_2^2 + \frac{1}{2\tau}\|u-w\|_2^2 \\ &= (\tau H^\top H + \mathrm{Id})^{-1}(\tau H^\top Hf + u),\end{aligned} \tag{33}$$

where the symbol $^\top$ denotes the adjoint of a linear operator and $H^\top : \mathbb{R}^N \to \mathbb{R}^N$ satisfies $\langle Hw, w'\rangle_{\mathbb{R}^N} = \langle w, H^\top w'\rangle_{\mathbb{R}^N}$. The evaluation of $\mathrm{prox}_{\tau\mathscr{H}}(u)$ corresponds to the inverse of a linear system that is not always possible in practice due to the high dimensionality of the problem.

For this reason, the optimization methods which involve the gradient of $\mathscr{H}$ are more appropriate since they do not require any inverse operator. In the comparison with $\mathscr{F}$ and $\mathscr{H}$, the calculation of the proximity operator in the case of the composite function $\mathscr{G} \circ L(u) = \lambda \|D_{2,\rho} u\|_2$ is theoretically possible but is challenging because of the presence of $D_{2,\rho}$ which is not diagonal.

To solve the minimization problem (29), we adopt the full splitting approach described in[28,29]. The key idea of this approach is to evaluate the gradient, proximity and linear operators individually in order to avoid implicit operations such as inner loops or inverse of linear operators. Accordingly, only "simple" computations are considered such as the gradient $\nabla\mathscr{H}$, the proximity operator of $\mathscr{F}$ and $\mathscr{G}$, the linear mapping L and its adjoint operators $L^\top$. The corresponding proximal algorithm for the problem is written under the following general form at iteration ℓ:

$$u^{(\ell+1)} = \mathrm{prox}_{\gamma\mathscr{F}}\left\{u^{(\ell)} - \gamma\left(L^\top z^{(\ell)} + \nabla\mathscr{H}(u^{(\ell)})\right)\right\}, \tag{34a}$$

$$z^{(\ell+1)} = \mathrm{prox}_{\zeta\mathscr{G}^\star}\left\{z^{(\ell)} + \zeta L\left(2u^{(\ell+1)} - u^{(\ell)}\right)\right\}, \tag{34b}$$

where $\gamma, \zeta > 0$ are proximal parameters, $\mathscr{G}^\star$ denotes the Legendre-Fenchel conjugate of $\mathscr{G}$ and its proximity operator $\mathrm{prox}_{\zeta\mathscr{G}^\star}$ can be directly computed from $\mathrm{prox}_{\frac{\mathscr{G}}{\zeta}}$ by using the Moreau's identity $v = \zeta\,\mathrm{prox}_{\frac{\mathscr{G}}{\zeta}}\left(\frac{v}{\zeta}\right) + \mathrm{prox}_{\zeta\mathscr{G}^\star}(v)$. Following[28,29], to guarantee the convergence of the proposed algorithm, the parameters γ and ζ must fulfill the condition

$$\gamma\left(\frac{1}{2} + \zeta |\!|\!| L^\top L |\!|\!|\right) < 1, \tag{35}$$

where $|\!|\!|\cdot|\!|\!|$ denotes the operator norm. The proofs of the convergence can be found in[28]. We also note that the proposed algorithm belongs to the class of primal-dual algorithms which provide not only the solution of the primal problem (a.k.a. the original minimization problem) but also the solution of its dual problem.

Since the closed-form of $\mathrm{prox}_{\gamma\mathscr{F}}$ is already given, it remains to define the analytic expression of other terms in (34a) and (34b). We start with the gradient of the quadratic function $\mathscr{H}$ which is straightforwardly obtained by

$$\nabla\mathscr{H}(u) = H^\top(Hu - f). \tag{36}$$

Next, we notice that the regularization operator $L = D_{2,\rho}$ is a linear mapping, then its adjoint operator $L^\top = D_{2,\rho}^\top$ is defined using the equation $\langle u, D_{2,\rho}^\top v\rangle = \langle D_{2,\rho} u, v\rangle$, which implies ($d = 3$)

$$\begin{aligned}\left(D_{2,\rho}{}^\top v\right)_{i,j,k} \quad = \quad & (1-\rho)v^1_{i,j,k} + \rho[(\Delta^{(2)}_{1,1})^\top v^2]_{i,j,k} + \rho[(\Delta^{(2)}_{2,2})^\top v^3]_{i,j,k} + \rho[(\Delta^{(2)}_{3,3})^\top v^4]_{i,j,k} \\ & + \rho[(\Delta^{(2)}_{1,2})^\top v^5]_{i,j,k} + \rho[(\Delta^{(2)}_{1,3})^\top v^6]_{i,j,k} + \rho[(\Delta^{(2)}_{2,3})^\top v^7]_{i,j,k},\end{aligned} \tag{37}$$

where the involving adjoint operators are given below:

$$[(\Delta^{(2)}_{1,1})^\top w]_{i,j,k} = \begin{cases} w_{i+1,j,k} - 2w_{i,j,k} + w_{i-1,j,k} & \text{if } 1 < i < N_x \\ 0 & \text{otherwise} \end{cases}$$

$$[(\Delta^{(2)}_{2,2})^\top w]_{i,j,k} = \begin{cases} w_{i,j+1,k} - 2w_{i,j,k} + w_{i,j-1,k} & \text{if } 1 < j < N_y \\ 0 & \text{otherwise} \end{cases}$$

$$[(\Delta^{(2)}_{3,3})^\top w]_{i,j,k} = \begin{cases} w_{i,j,k+1} - 2w_{i,j,k} + w_{i,j,k-1} & \text{if } 1 < k < N_z \\ 0 & \text{otherwise} \end{cases}$$

$$[(\Delta^{(2)}_{1,2})^\top w]_{i,j,k} = [(\Delta^{(2)}_{2,1})^\top w]_{i,j,k} =$$
$$\begin{cases} w_{i,j,k} & \text{if } i = 1, j = 1, 1 \le k \le N_z \\ w_{i,j,k} - w_{i,j-1,k} & \text{if } i = 1, 1 < j < N_y, 1 \le k \le N_z; \\ -w_{i,j-1,k} & \text{if } i = 1, j = N_y, 1 \le k \le N_z; \\ & \\ w_{i,j,k} - w_{i-1,j,k} & \text{if } 1 < i < N_x, j = 1, 1 \le k \le N_z; \\ w_{i,j,k} - w_{i-1,j,k} - w_{i,j-1,k} + w_{i-1,j-1,k} & \text{if } 1 < i < N_x, 1 < j < N_y, 1 \le k \le N_z; \\ -w_{i,j-1,k} + w_{i-1,j-1,k} & \text{if } 1 < i < N_x, j = N_y, 1 \le k \le N_z; \\ & \\ -w_{i-1,j,k} & \text{if } i = N_x, j = 1, 1 \le k \le N_z; \\ -w_{i-1,j,k} + w_{i-1,j-1,k} & \text{if } i = N_x, 1 < j < N_y, 1 \le k \le N_z; \\ w_{i-1,j-1,k} & \text{if } i = N_x, j = N_y, 1 \le k \le N_z. \end{cases}$$

$$[(\Delta_{1,3}^{(2)})^\top w]_{i,j,k} = [(\Delta_{3,1}^{(2)})^\top w]_{i,j,k} =$$

$$\begin{cases} w_{i,j,k} & \text{if } i=1, k=1, 1 \le j \le N_y \\ w_{i,j,k} - w_{i,j,k-1} & \text{if } i=1, 1<k<N_z, 1 \le j \le N_y; \\ -w_{i,j,k-1} & \text{if } i=1, k=N_z, 1 \le j \le N_y; \\ & \\ w_{i,j,k} - w_{i-1,j,k} & \text{if } 1<i<N_x, k=1, 1 \le j \le N_y; \\ w_{i,j,k} - w_{i-1,j,k} - w_{i,j,k-1} + w_{i-1,j,k-1} & \text{if } 1<i<N_x, 1<k<N_z, 1 \le j \le N_y; \\ -w_{i,j,k-1} + w_{i-1,j,k-1} & \text{if } 1<i<N_x, k=N_z, 1 \le j \le N_y; \\ & \\ -w_{i-1,j,k} & \text{if } i=N_x, k=1, 1 \le j \le N_y; \\ -w_{i-1,j,k} + w_{i-1,j,k-1} & \text{if } i=N_x, 1<k<N_z, 1 \le j \le N_y; \\ w_{i-1,j,k-1} & \text{if } i=N_x, k=N_z, 1 \le j \le N_y. \end{cases}$$

$$[(\Delta_{2,3}^{(2)})^\top w]_{i,j,k} = [(\Delta_{3,2}^{(2)})^\top w]_{i,j,k} =$$

$$\begin{cases} w_{i,j,k} & \text{if } j=1, k=1, 1 \le i \le N_x \\ w_{i,j,k} - w_{i,j,k-1} & \text{if } j=1, 1<k<N_z, 1 \le i \le N_x; \\ -w_{i,j,k-1} & \text{if } j=1, k=N_z, 1 \le i \le N_x; \\ & \\ w_{i,j,k} - w_{i,j-1,k} & \text{if } 1<j<N_y, k=1, 1 \le i \le N_x; \\ w_{i,j,k} - w_{i,j-1,k} - w_{i,j,k-1} + w_{i,j-1,k-1} & \text{if } 1<j<N_y, 1<k<N_z, 1 \le i \le N_x; \\ -w_{i,j,k-1} + w_{i,j-1,k-1} & \text{if } 1<j<N_y, k=N_z, 1 \le i \le N_x; \\ & \\ -w_{i,j-1,k} & \text{if } j=N_y, k=1, 1 \le i \le N_x; \\ -w_{i,j-1,k} + w_{i,j-1,k-1} & \text{if } j=N_y, 1<k<N_z, 1 \le i \le N_x; \\ w_{i,j-1,k-1} & \text{if } j=N_y, k=N_z, 1 \le i \le N_x. \end{cases}$$

From equations (37), one can deduce the following upper bound:

$$|||D_{2,\rho}{}^\top D_{2,\rho}||| \le (1-\rho)^2 + 64\rho^2 \,, \tag{38a}$$

that are used for choosing the proximal parameters γ and ζ according to (35).

The last term we want to deal with is the proximity operator $\text{prox}_{\zeta\mathscr{G}^\star}$. We also note that the proposed primal-dual algorithm does not necessitate evaluating the proximity operator of the composite function $\mathscr{G} \circ L$ as in the case of generic proximal algorithms, but only $\text{prox}_{\frac{\mathscr{G}}{\zeta}}$ is required. Since $\mathscr{G}$ is related to the mixed norm whose the proximity operator is defined as:

$$\left(\text{prox}_{\tau\|\cdot\|_2}(v)\right)^\kappa_{i,j,k} = \max(0, \|L_{i,j,k} v\|_2 - \tau)\frac{v^\kappa_{i,j,k}}{\|L_{i,j,k} v\|_2} \,, \tag{39}$$

where $L_{i,j,k} : v \in \mathscr{X}^{10} \mapsto (v^\kappa_{i,j,k})_{1\le\kappa\le 10} \in \mathbb{R}^{10}$ is a linear operator ($d=3$). By using the Moreau's identity, we obtain the closed-form expression of $\text{prox}_{\zeta\mathscr{G}^\star}$ as the following:

$$\left(\text{prox}_{\zeta\mathscr{G}^\star}(v)\right)^\kappa_{i,j,k} = \frac{v^\kappa_{i,j,k}}{\max\left(1, \frac{\|L_{i,j,k} v\|_2}{\lambda}\right)} \,, \tag{40}$$

which shows that $\text{prox}_{\zeta\mathscr{G}^\star}$ is independent from ζ and moreover it is an pointwise operator. These properties allow therefore fast and efficient computation by exploiting the intrinsic parallelism of multicore processors.

Energy model for 4D denoising

A temporal series of 3D noisy images $f : \Omega \subset \mathbb{R}^4 \to \mathbb{R}$ is a noisy version of the underlying true 3D image sequence $u : \Omega \to \mathbb{R}$ modeled as follows: $f = \mathscr{T}(u)$. It follows that the denoising problem is formulated as the minimization of an energy functional defined as

$$\int_\Omega (u(\mathbf{x}) - f(\mathbf{x}))^2 \,+\, \lambda \|D_{2,\rho} u(\mathbf{x})\|_2 \, d\mathbf{x} \,+\, \iota_{\mathscr{C}}(u) \tag{41}$$

where

$$
\begin{aligned}
D_{2,\rho}u(\mathbf{x}) \quad := \quad & ((1-\rho)u(\mathbf{x}), \\
& \rho\ \frac{\partial^2 u(\mathbf{x})}{\partial_{xx}},\ \rho\ \frac{\partial^2 u(\mathbf{x})}{\partial_{yy}},\ \rho\ \frac{\partial^2 u(\mathbf{x})}{\partial_{zz}},\ \rho\ \frac{\partial^2 u(\mathbf{x})}{\partial_{tt}},\ \rho\ \frac{\partial^2 u(\mathbf{x})}{\partial_{xy}},\ \rho\ \frac{\partial^2 u(\mathbf{x})}{\partial_{xz}},\ \rho\ \frac{\partial^2 u(\mathbf{x})}{\partial_{xt}}, \\
& \rho\ \frac{\partial^2 u(\mathbf{x})}{\partial_{yx}},\ \rho\ \frac{\partial^2 u(\mathbf{x})}{\partial_{yz}},\ \rho\ \frac{\partial^2 u(\mathbf{x})}{\partial_{yt}},\ \rho\ \frac{\partial^2 u(\mathbf{x})}{\partial_{zx}},\ \rho\ \frac{\partial^2 u(\mathbf{x})}{\partial_{zy}},\ \rho\ \frac{\partial^2 u(\mathbf{x})}{\partial_{zt}}\ \Big) \in \mathbb{R}^{14}\ .
\end{aligned}
\tag{42}
$$

To solve the optimization problem, we split the original problem (41) into several simple sub-problems as explained above. The resulting individual functions involved in the sum are then minimized separately. The algorithm is expected to be faster since FFT is not required in the implementation for image denoising. Nevertheless, the amount of data is much more larger since the whole 4D image sequence is denoised at once. In practice, a very long image sequence can be segmented into sub-sequences with a small overlap, and is processed independently and in parallel. The overlapping images can be averaged to reduce possible artifacts at the end.

SUPPLEMENTARY FIGURES

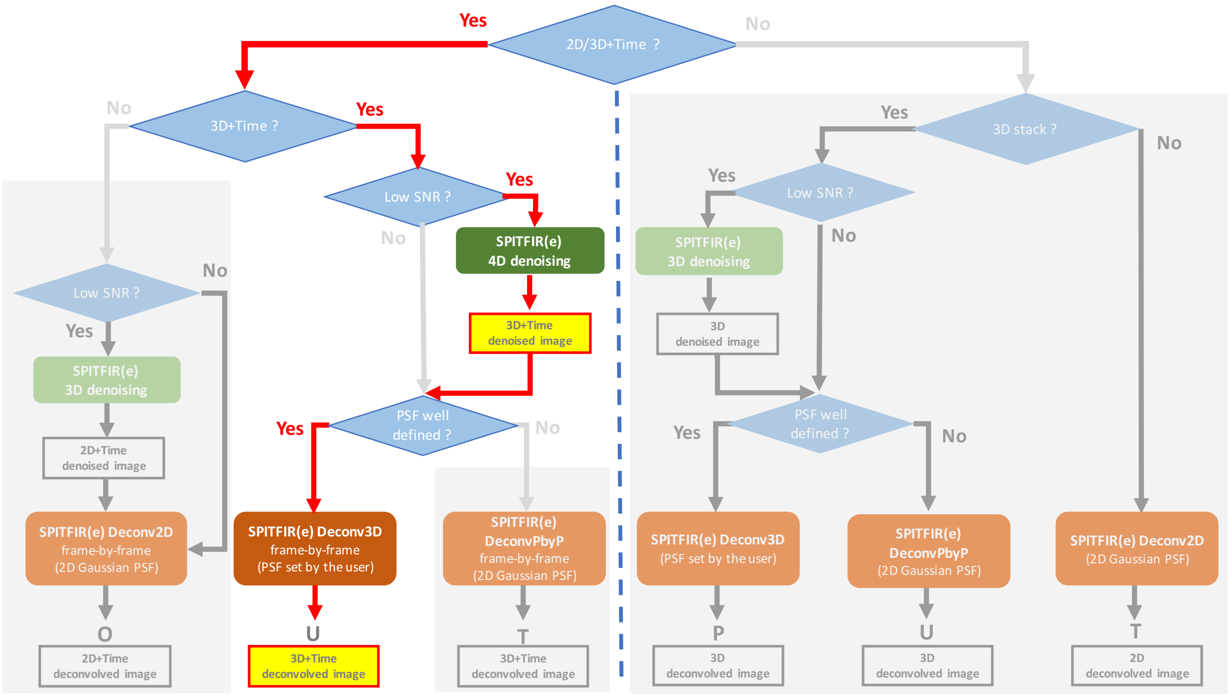

Supplementary Figure S1. **Workflow for image restoration extracted from the global flow chart in Fig. 1 applied to 4D-denoising, background subtraction, and 3D deconvolution of mitochondria and microtubules images shown in Fig. 2.**

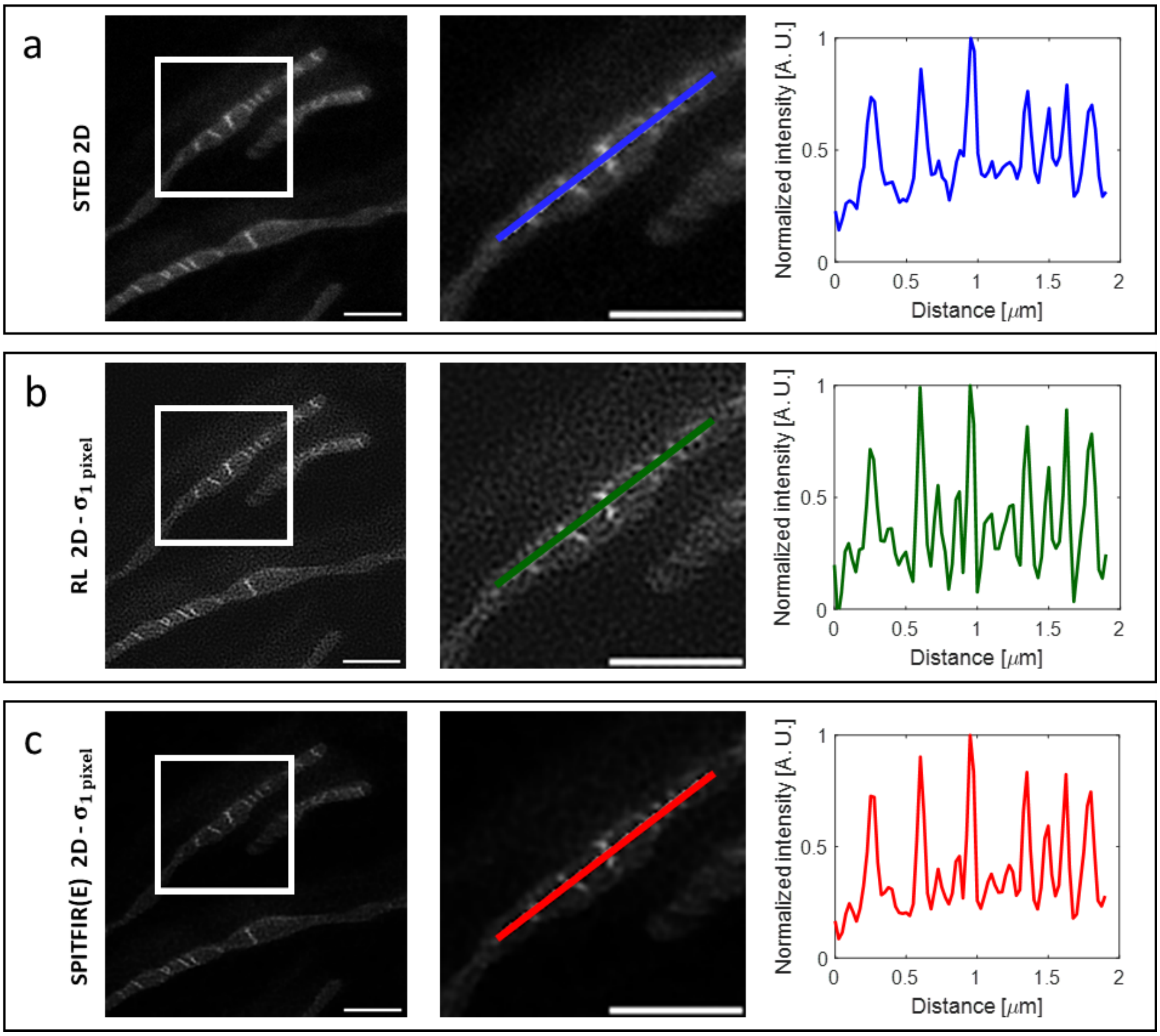

Supplementary Figure S2. Comparison of 2D image improvement in STED imaging. Images of Mitochondria (PKMO) in Live RPE1 cells (used in Fig. 3) before (**a**), after being processed by Richardson-Lucy 2D deconvolution (**b**) and SPITFIR(e) 2D denoising + 2D deconvolution (**c**). (**b**) Richardson-Lucy(RL) 3D deconvolution using `deconvlucy` from MATLAB imaging processing toolbox (2D Gaussian PSF and $\sigma_{xy} = 1$ pixel; 10 iterations). (**c**) SPITFIR(e) 2D denoising + 2D deconvolution (2D Gaussian PSF and $\sigma_{xy} = 1$ pixel). Insets are zoomed area illustrating SPITFIR(e) improvement (**b**; center) in signal to noise ratio (SNR) and image quality as compared to RL treatment (**c**; center). Intensity line profiles were measured as indicated in the insets and plotted for 2D STED (**a**; right), RL (**b**; right) and SPITFIRE(e) (**c**; left). Scale bars = 1 μm.

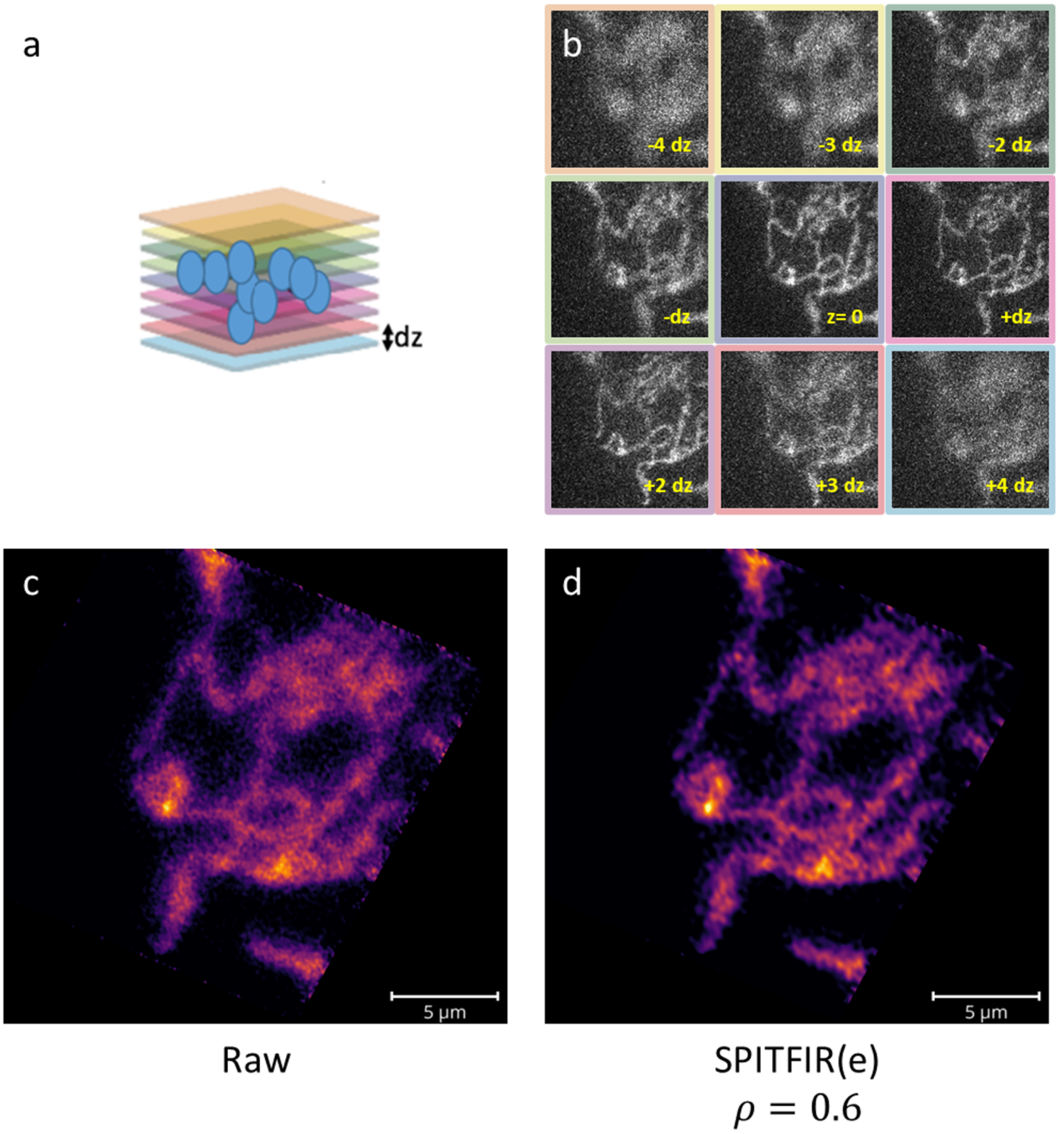

Supplementary Figure S3. 3D multifocus microscopy reconstruction using SPITFIR(e). Data are temporal series (100 time points) of 3D stacks composed of nine planes each depicting mitochondria in U2OS cells transfected with TOM20 (translocase of outer mitochondrial membrane) fused to GFP (GFP-TOM20). Exposure time: 50 ms. 3D MFM stack (**a**) is acquired at the same time point. (**b**) Z-planes are equally spaced with dz = 330 nm and pixel size = 120 nm. 3D angular views are shown before (**c**) and after (**d**) SPITFIR(e) using "moderate" sparsity level. 3D average rendering was performed in napari. Data corresponds to EXP 2 on Fig. 4 (**a**). Scale bar: 5 μm.

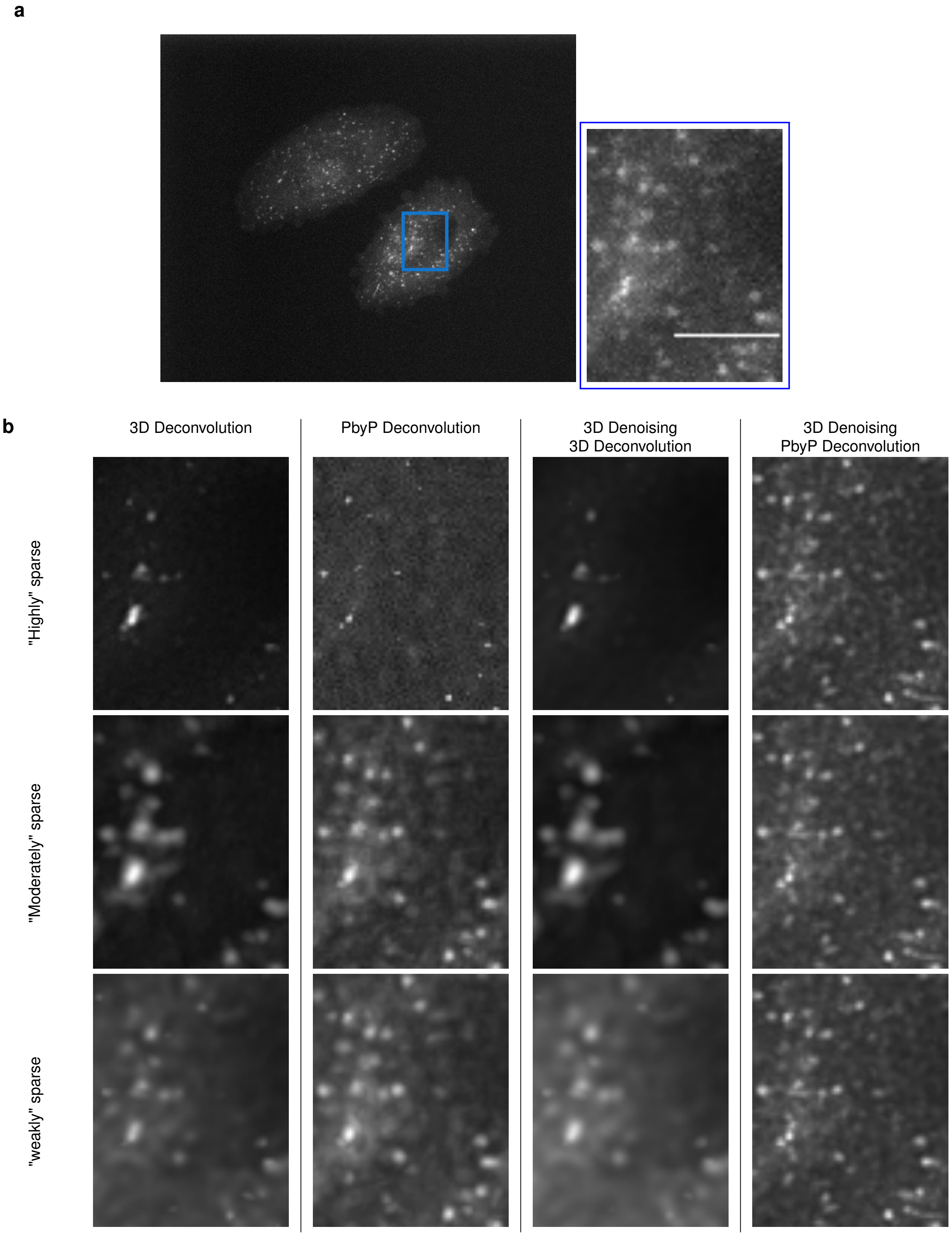

Supplementary Figure S4. Deconvolution of 3D spinning-disk confocal microscopy volume (14 planes) depicting live Hela cells expressing Rab5-eGFP proteins (exposure time: 100 ms). (**a**) Display of the maximum intensity projection along the Z-axis of raw data (scale bar: 8 μm). (**b**) Results with SPITFIR(e) with different amounts of sparsity ("weak" ($\rho = 0.9$), "moderate" ($\rho = 0.6$), "high" ($\rho = 0.1$), automatic selection of the regularization parameter, and different strategies with SPITFIR(e): 3D Deconvolution, PbyP Deconvolution, 3D Denoising + 3D Deconvolution, 3D Denoising + PbyP deconvolution ($\sigma_{xy} = 1.5$ pixels and $\sigma_z = 0.5$ pixel).

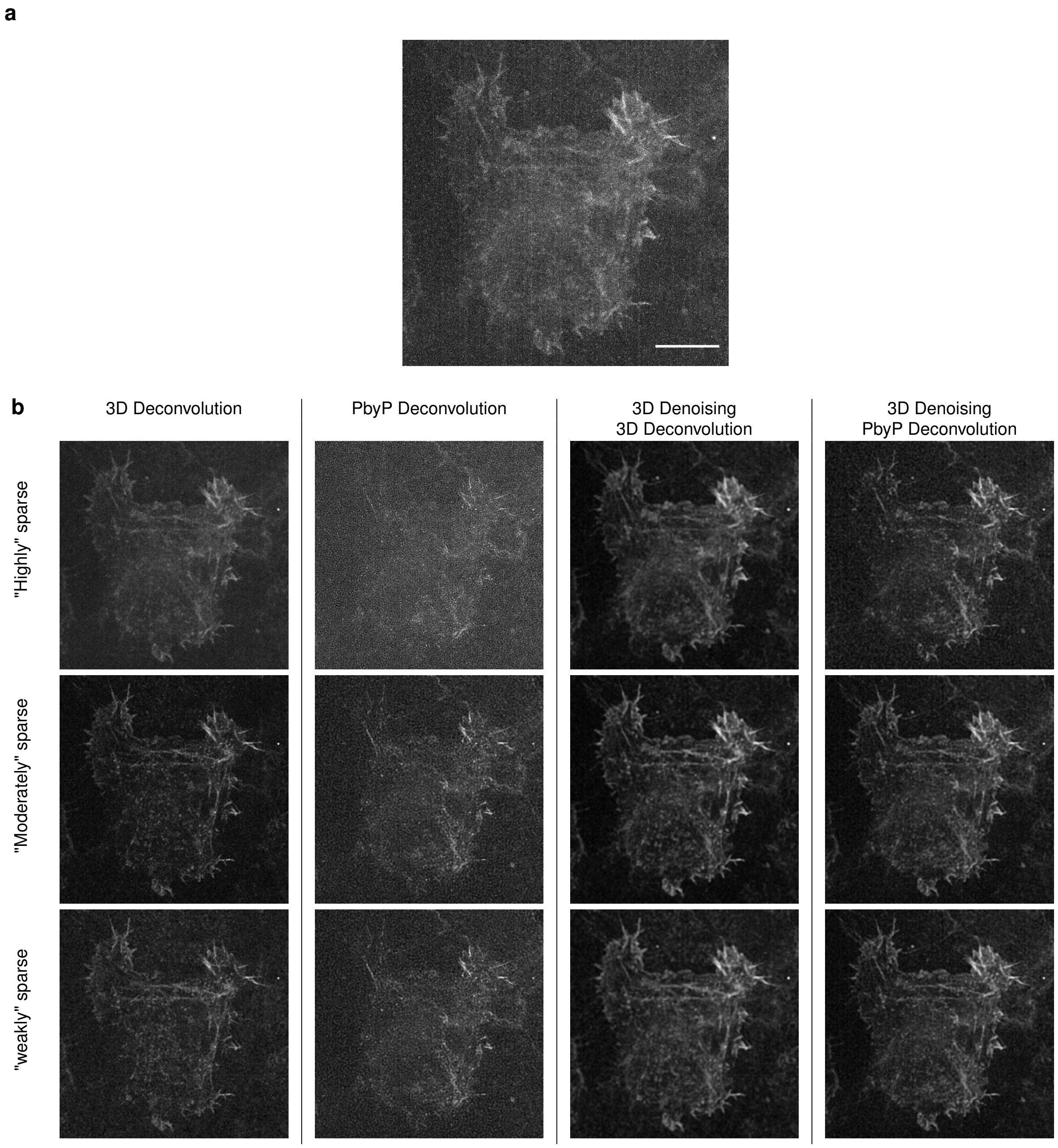

Supplementary Figure S5. Deconvolution of 3D spinning-disk confocal microscopy volume (24 planes) depicting live RPE1 cells expressing mCherry-LifeAct (exposure time: 20 ms). (**a**) Display of the maximum intensity projection along the Z-axis of raw data (scale bar: 10 μm). (**b**) Results with SPITFIR(e) with different levels of sparsity ("weak" ($\rho = 0.9$), "moderate" ($\rho = 0.6$), "high" ($\rho = 0.1$), automatic selection of the regularization parameter and different strategies with SPITFIR(e): 3D Deconvolution, PbyP Deconvolution, 3D Denoising + 3D Deconvolution, 3D Denoising + PbyP deconvolution ($\sigma_{xy} = 1.5$ pixels and $\sigma_z = 0.5$ pixel).

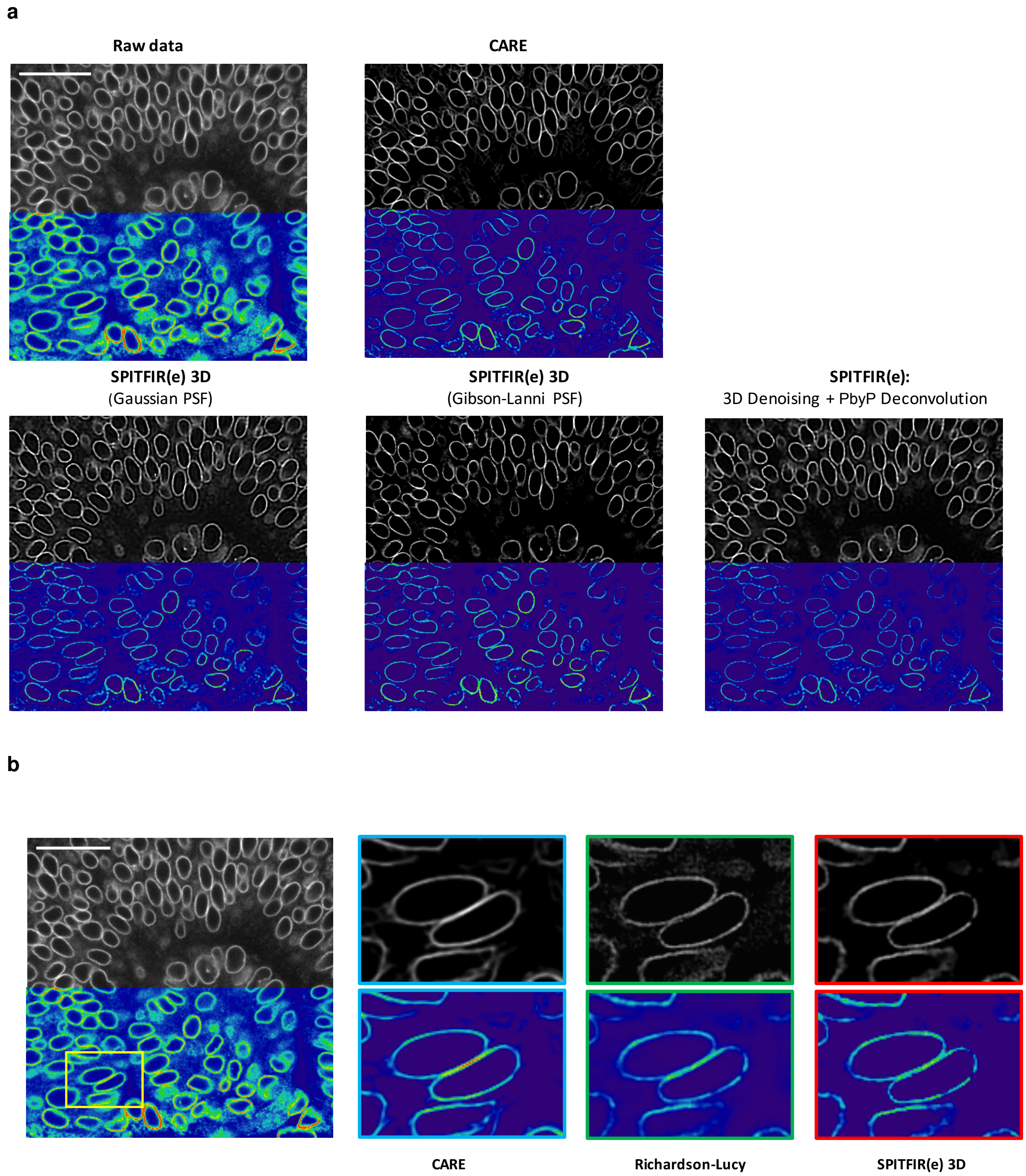

Supplementary Figure S6. Deconvolution of a 3D spinning-disk confocal microscopy image depicting the envelopes of nuclei stained with GFP-LAP2b (developing eye of zebrafish (Daniorerio) embryos, 60X/1.3-NA objective, 488 nm, z step = 2 μm, source:[43]). (**a**) Comparison of CARE and SPITFIR(e) (3D deconvolution with a Gibson-Lanni PSF model (generated from[43]), 3D deconvolution with a 3D Gaussian PSF model ($\sigma_{xy} = 2.0$ pixels and $\sigma_z = 0.5$ pixel), 3D denoising + PbyP deconvolution ($\sigma_{xy} = 2.0$ pixels)). We display the results obtained on the 5th plane (scale bar: 25 μm). (**b**) Zoom-in views (5th plane) of deconvolution results obtained with CARE, RL, and SPITFIR(e) 3D (Gibson-Lanni PSF model).
